# The *Arabidopsis thaliana* Double-Stranded RNA Binding Proteins DRB1 and DRB2 Are Required for miR160-Mediated Responses to Exogenous Auxin

**DOI:** 10.3390/genes15121648

**Published:** 2024-12-21

**Authors:** Kim Zimmerman, Joseph L. Pegler, Jackson M. J. Oultram, David A. Collings, Ming-Bo Wang, Christopher P. L. Grof, Andrew L. Eamens

**Affiliations:** 1 Centre for Plant Science, School of Environmental and Life Sciences, College of Engineering, Science and Environment University of Newcastle, Callaghan, NSW 2308, Australia; kim.zimmerman3@det.nsw.edu.au (K.Z.); joseph.pegler@newcastle.edu.au (J.L.P.); jackson.oultram@newcastle.edu.au (J.M.J.O.); david.collings@anu.edu.au (D.A.C.); chris.grof@newcastle.edu.au (C.P.L.G.); 2 Research School of Biology, Australian National University, Canberra, ACT 2601, Australia; 3 CSIRO Agriculture and Food, Canberra, ACT 2601, Australia; ming-bo.wang@csiro.au; 4 School of Agriculture and Food Sustainability, The University of Queensland, St. Lucia, QLD 4072, Australia; 5 Seaweed Research Group, School of Health, University of the Sunshine Coast, Maroochydore, QLD 4558, Australia

**Keywords:** *Arabidopsis thaliana* (*Arabidopsis*), root development, microRNA160 (miR160), miR160 expression module, *AUXIN RESPONSE FACTOR10* (*ARF10*), *ARF16*, *ARF17*, synthetic auxin

## Abstract

DOUBLE-STRANDED RNA BINDING (DRB) proteins DRB1, DRB2, and DRB4 are essential for microRNA (miRNA) production in *Arabidopsis thaliana* (*Arabidopsis*) with miR160, and its target genes, *AUXIN RESPONSE FACTOR10* (*ARF10*), *ARF16*, and *ARF17*, forming an auxin responsive miRNA expression module crucial for root development. **Methods**: Wild-type *Arabidopsis* plants (Columbia-0 (Col-0)) and the *drb1*, *drb2*, and *drb12* mutants were treated with the synthetic auxin 2,4-dichlorophenoxyacetic acid (2,4-D), and the miR160-mediated response of these four *Arabidopsis* lines was phenotypically and molecularly characterized. **Results**: In 2,4-D-treated Col-0, *drb1* and *drb2* plants, altered miR160 abundance and *ARF10*, *ARF16*, and *ARF17* gene expression were associated with altered root system development. However, miR160-directed molecular responses to treatment with 2,4-D was largely defective in the *drb12* double mutant. In addition, via profiling of molecular components of the miR160 expression module in the roots of the *drb4*, *drb14*, and *drb24* mutants, we uncovered a previously unknown role for DRB4 in regulating miR160 production. **Conclusions**: The miR160 expression module forms a central component of the molecular and phenotypic response of *Arabidopsis* plants to exogenous auxin treatment. Furthermore, DRB1, DRB2, and DRB4 are all required in *Arabidopsis* roots to control miR160 production, and subsequently, to appropriately regulate *ARF10*, *ARF16*, and *ARF17* target gene expression.

## 1. Introduction

The *AUXIN*/*INDOLE-3-ACETIC ACID* (*Aux*/*IAA*), *TRANSPORT INHIBITOR RESPONSE* (*TIR*), and *AUXIN RESPONSE FACTOR* (*ARF*) gene families form the three main gene families that direct the molecular responses of *Arabidopsis thaliana* (*Arabidopsis*) to auxin signaling and the intracellular perception of auxin [1,2,3,4,5]. ARF function is repressed in the absence of auxin by their interaction with specific members of the Aux/IAA protein family [2,3,4]. The ARF protein family contains 23 members in *Arabidopsis*, of which five function as transcriptional activators, with the remaining 18 functioning as transcriptional repressors [2]. The transcription factor function of each ARF is determined by three regions of each protein, including the N-terminal DNA-binding domain (DBD), an activator (AD) or repressor domain (RD) positioned in the central region of the protein, and the dimerization domain (CTD) located at the C-terminal region [6,7]. The CTD facilitates the formation of ARF:Aux/IAA heterodimers, and directs the ARF:ARF dimerization required for the transcription factor function of each ARF [6,7,8]. The DBD mediates the interaction of ARF protein dimers with the promoter of each *AUXIN RESPONSE GENE* (*ARG*), where the AD or RD locate the *cis*-acting *AUXIN RESPONSE ELEMENT*s (*ARE*s) to direct either *ARG* transcription activation or repression [6,7].

Through transcriptional control of *ARG* activity, ARFs play numerous and functionally diverse roles in plant development. For example, ARF5 and ARF7, both transcriptional activators, are major players in embryonic patterning [9]. Furthermore, ARF5, independent of ARF7, regulates primary root initiation [10]. This underlines the importance of ARFs from the earliest stages of plant development. In addition, ARF1 and ARF2 exemplify the importance of ARF-directed regulation of *ARG* expression throughout *Arabidopsis* development, as they function together to regulate leaf senescence and floral organ abscission [11], processes important for the later stages of vegetative development, and ultimately, the final stage of the *Arabidopsis* life cycle, reproduction. Given their importance in *Arabidopsis* development, it is unsurprising that several *ARF* gene family members are themselves under additional regulation at the molecular level. Specifically, (1) microRNA160 (miR160) post-transcriptionally regulates *ARF10*, *ARF16*, and *ARF17* expression [12,13]; (2) miR167 controls *ARF6* and *ARF8* transcript abundance [14,15]; and (3) *ARF2*, *ARF3*, and *ARF4* are regulated at the posttranscriptional level by tasiARF, a *trans*-acting small-interfering RNA (tasiRNA) [16]. The small RNA (sRNA)-regulated *ARF* genes in each of the regulated subclades show a close phylogenetic relationship and those within each subclade have closely related functions. For example, ARF2, ARF3, and ARF4 all function to determine the polarity of leaves or other organs [11,17]. The two miR167 targets, ARF6 and ARF8, have an even closer relationship, functioning redundantly in regulating flower maturation [14,17,18]. The miR160-regulated family members, ARF10, ARF16, and ARF17, play a role in regulating root cap development, lateral root primordia formation, and root growth [12,13]. The ARF10 and ARF16 proteins function either in concert with each other or redundantly, to control root development [13]. *ARF17* encodes a protein that is truncated at its C-terminus [2], which likely accounts for its functional divergence from *ARF10* and *ARF16*.

Like miR160 and miR167, numerous other miRNAs play central roles in regulating the expression of developmentally important genes in *Arabidopsis* [19,20,21]. Each miRNA is processed from a stem-loop structured double-stranded RNA (dsRNA) precursor by the RNase III-like endonuclease DICER-LIKE1 (DCL1) in conjunction with a dsRNA BINDING (DRB) protein. *Arabidopsis* encodes five DRBs, of which DRB1 functions with DCL1 to produce most *Arabidopsis* miRNAs [22,23,24]. Specifically, DRB1 positions DCL1 on the stem-loop of the miRNA precursor to ensure accurate processing [25,26,27]. miRNA production via the DRB1/DCL1 functional partnership occurs via a two-step process: (1) DCL1 cleaves off the unpaired 5′ and 3′ regions of the primary-miRNA (pri-miRNA) to leave only the stem-loop structured region termed the precursor-miRNA (pre-miRNA); and (2) DCL1 next removes the loop and part of the stem arms of the pre-miRNA to liberate the miRNA/miRNA* duplex [23,25,26,27]. DRB1 then orientates the miRNA/miRNA* duplex for its directional loading into the ARGONAUTE1 (AGO1) endonuclease for AGO1-catalyzed cleavage of the miRNA* passenger strand [28]. AGO1 retains the miRNA guide strand to form the miRNA-induced silencing complex (miRISC), which directs the expression regulation of target gene transcripts that have high sequence complementarity to the loaded miRNA [29,30,31,32].

While DRB1 is the primary DRB protein involved in miRNA production in *Arabidopsis*, two other DRB proteins, DRB2 and DRB4, also play roles in miRNA production [33,34,35,36]. DRB4 interacts with DCL4 to process the non-conserved (or newly evolved) subclass of *Arabidopsis* miRNAs from highly structured stem-loop precursors which have extensive dsRNA base-pairing between the two stem arms and minimal loop regions, structural features recognized by DRB4 [28,33,34]. Like DRB1 and DCL1, DRB4 accurately positions DCL4 on these specifically structured precursors to ensure efficient processing of the non-conserved miRNA subclass [33,34]. Unlike DRB1 and DRB4, which interact exclusively with DCL1 and DCL4, respectively, DRB2 appears to be able to functionally interact with both DCL endonucleases, with this DRB2 interaction likely disrupting the canonical DRB1/DCL1 and DRB4/DCL4 functional partnerships [28,34,35,36,37]. However, the involvement of DRB2 in miRNA production in *Arabidopsis* is limited to specific miRNA subsets due to the expression of *DRB2* being restricted to developmentally important tissues [35,36,37,38].

In this study, we investigated the roles of DRB proteins in the miR160/*ARF10*/*ARF16*/*ARF17* expression module in *Arabidopsis* root system development following the application of the synthetic auxin 2,4-dichlorophenoxyacetic acid (2,4-D). We first examined the phenotypic changes of wild-type *Arabidopsis* plants (ecotype Columbia-0 (Col-0)) and the *drb1*, *drb2*, and *drb12* mutants, and studied the expression profiles of the various components in the miR160 expression module after 2,4-D treatment. These experiments revealed altered miR160 accumulation and *ARF10*, *ARF16*, and *ARF17* gene expression in Col-0, *drb1*, and *drb2* plants after 2,4-D treatment, molecular changes which were associated with the altered root system development observed in these three *Arabidopsis* lines. However, such molecular and phenotypic responses to 2,4-D were largely defective in the *drb12* double mutant. In addition, molecular profiling of all components of the miR160 expression module in the *drb4*, *drb14*, and *drb24* mutant backgrounds revealed a previously unknown role for DRB4 in supplying an additional layer of regulatory complexity to the miR160 expression module. Together, our results show that DRB1, DRB2, and DRB4 all play a role in regulating miR160 production to subsequently ensure tight control of miR160-directed regulation of *ARF10*, *ARF16*, and *ARF17* expression, and to thus guarantee normal development of the *Arabidopsis* root system.

## 2. Materials and Methods

### 2.1. Plant Lines, Growth Conditions, and Phenotypic Assessments

The *Arabidopsis thaliana* (*Arabidopsis*) ecotype Columbia-0 (Col-0) was used as the wild-type control for all experiments and the *drb* single mutants, *drb1-1* (*drb1*; SALK_064863), *drb2-1* (*drb2*; GABI_348A09), and *drb4-1* (*drb4*; SALK_000736), which harbor a T-DNA insertion mutation in the *DRB1* (*AT1G09700*), *DRB2* (*AT2G28380*), and *DRB4* (*AT3G62800*) locus, respectively, are also in the Col-0 background, and have been described previously [28,34,38]. The double mutant lines used in this study, specifically the *drb1-1 drb4-1* (*drb14*), and *drb2-1 drb4-1* (*drb24*) double mutants, were generated via standard genetic crossing [34,35,37,38]. Seed sterilization, stratification, and germination, and subsequent plant growth were exactly as described in our previous study [39]. At day 10, healthy Col-0, *drb1*, *drb2*, and *drb12* seedlings were transferred to fresh MS growth medium which had been supplemented with either 0.0 (untreated controls), 0.1, or 1.0 micromolar (μM) of 2,4-D (Sigma Aldrich, Melbourne, Australia). Post seedling transfer, medium plates were resealed with gas permeable tape and returned to the temperature-controlled growth cabinet for 24 h. The 0.0, 0.1, and 1.0 μM 2,4-D treated seedlings were next transferred to fresh MS medium plates which contained standard plant growth medium; the plates were sealed with gas permeable tape, and then returned to the temperature-controlled growth cabinet. For this third period of cultivation, the plates were orientated vertically for a 10-day period under standard *Arabidopsis* growth conditions [39]. To investigate the potential regulatory involvement of DRB4 in the miR160 expression module, Col-0, *drb4*, *drb14*, and *drb24* seeds were sterilized, stratified, and cultivated on horizontally-orientated medium plates for a 10-day period, as outlined above for the other lines. At day 10, healthy seedlings were selected for transferal to fresh MS medium plates; the plates were sealed with gas permeable tape, and then returned to the temperature-controlled growth cabinet where they were orientated vertically for a further 11-day cultivation period under standard *Arabidopsis* growth conditions, as outlined in [39]. This additional day of cultivation post seedling transfer was included so that all the phenotypic and molecular analyses performed in this study were conducted on 21-day-old plants. At day 21, images of each of the seven *Arabidopsis* lines under assessment were captured and processed exactly as outlined in our previous study [39].

### 2.2. RNA Extraction, Complementary DNA Synthesis, and Gene Expression Analysis

For the molecular assessments, total RNA was extracted from three biological replicates of pooled root material sampled from six 21-day-old vertically grown seedlings of the plant lines Col-0, *drb1*, *drb2*, *drb4*, *drb12*, *drb14*, and *drb24*, using TRIzol^TM^ Reagent according to the protocol of the manufacturer (Thermo Fisher Scientific, Sydney, Australia). The synthesis of a miR160-specific complementary DNA (cDNA), or the synthesis of a high molecular weight cDNA library, was performed exactly as outlined in our previous study [39]. The quantification of miR160 abundance or the expression of each assessed gene was also conducted exactly as outlined in our previous study [39]. miR160 abundance and gene transcript expression were quantified using the 2^−∆∆CT^ method with the small nucleolar RNA, snoR101, and *ELONGATION FACTOR-1α* (*EF-1α*; *AT5G60390*) used as the respective internal controls to normalize the relative abundance of miR160 or the level of expression of each assessed gene. For all RT-qPCR experiments reported here, three biological replicates consisting of pools of six individual plants were used per sample, and three technical replicates were performed per biological replicate. The sequence of each DNA oligonucleotide used in this study, either for the synthesis of a miR160-specific cDNA, or to quantify gene transcript abundance via RT-qPCR, is provided in Table S1.

### 2.3. Statistical Analysis

**Figure 1:**
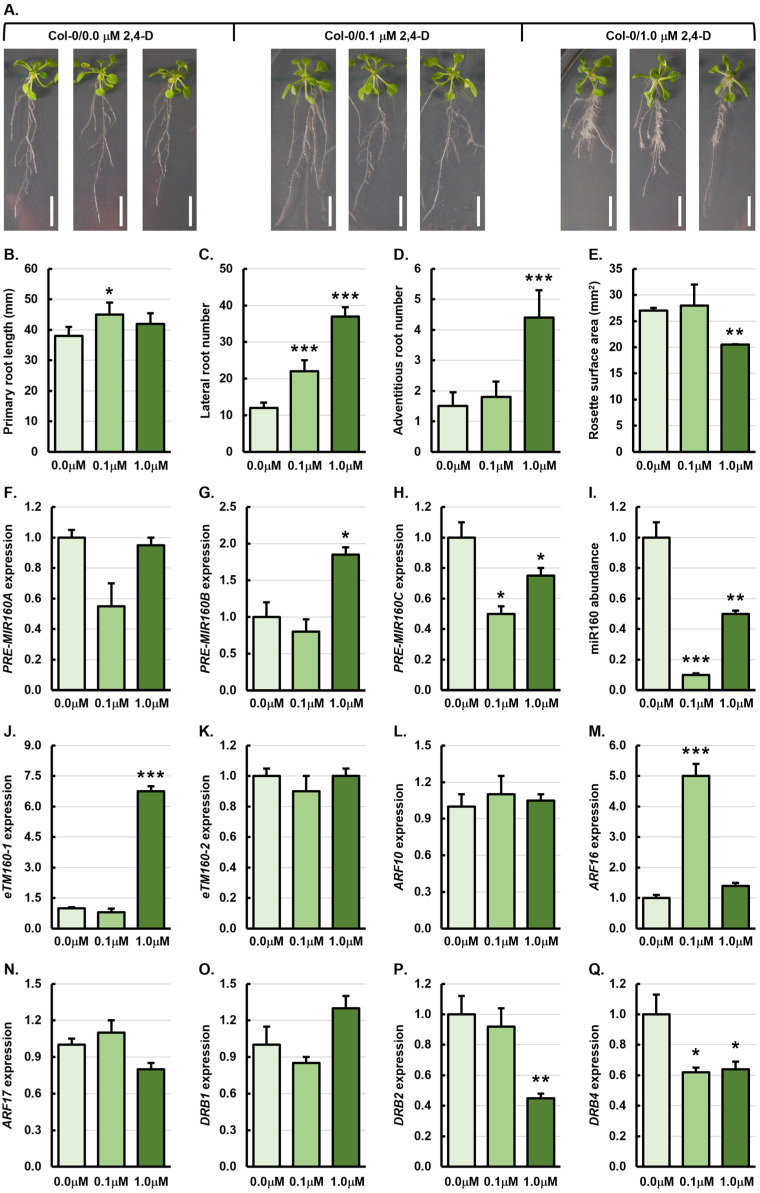
Phenotypic and molecular analysis of Col-0 plants treated with 2,4-D. (**A**) Representative phenotypes displayed by three individual 21-day-old untreated Col-0 plants or following the treatment of Col-0 plants with 0.1 μM and 1.0 μM 2,4-D. Scale bar = 1.0 cm. (**B**–**E**) Quantification of primary root length (**B**), lateral root number (**C**), adventitious root number (**D**), and rosette leaf surface area (**E**) of 21-day-old untreated Col-0 plants or following the treatment of Col-0 plants with 0.1 μM and 1.0 μM 2,4-D. (**F**–**Q**) Molecular analysis of all components of the miR160 expression module in the roots of 21-day-old untreated Col-0 plants or following the treatment of Col-0 plants with 0.1 μM and 1.0 μM 2,4-D, including profiling the expression of *PRE-MIR160A* (**F**), *PRE-MIR160B* (**G**), *PRE-MIR160C* (**H**), miR160 (**I**), *eTM160-1* (**J**), *eTM160-2* (**K**), *ARF10* (**L**), *ARF16* (**M**), *ARF17* (**N**), *DRB1* (**O**), *DRB2* (**P**), and *DRB4* (**Q**). (**B**–**Q**) All phenotypic and molecular analyses relied on the use of three biological replicates and each replicate consisted of a pool of six plants. Altered development (**B**–**E**) or changed transcript abundance (**F**–**Q**) was determined via comparison of the Col-0/0.1 μM and Col-0/1.0 μM treated samples to the values obtained for the untreated Col-0/0.0 μM sample by a standard two-tailed *t*-test. Error bars represent the standard error of the mean (SEM) and an asterisk (*) shows * *p* ≤ 0.05, ** *p* ≤ 0.01, *** *p* ≤ 0.001.

**Figure 2:**
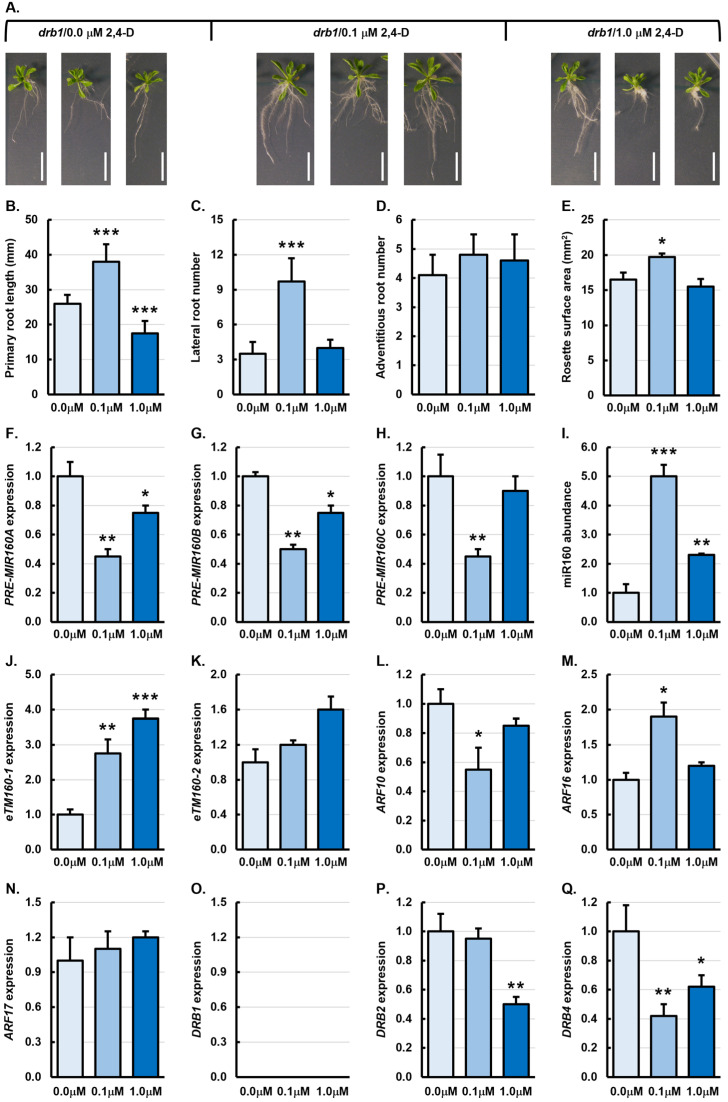
Phenotypic and molecular analysis of 21-day-old *drb1* plants treated with 2,4-D. (**A**) Representative phenotypes displayed by three individual 21-day-old untreated *drb1* control plants or following treatment of *drb1* plants with 0.1 μM and 1.0 μM 2,4-D. Scale bar = 1.0 cm. (**B**–**E**) Quantification of primary root length (**B**), lateral root number (**C**), adventitious root number (**D**), and rosette leaf surface area (**E**) of 21-day-old untreated *drb1* control plants or following the treatment of *drb1* plants with 0.1 μM and 1.0 μM 2,4-D. (**F**–**Q**) Molecular analysis of all components of the miR160 expression module in the roots of 21-day-old untreated *drb1* plants or following treatment of *drb1* plants with 0.1 μM and 1.0 μM 2,4-D, including profiling of the expression of *PRE-MIR160A* (**F**), *PRE-MIR160B* (**G**), *PRE-MIR160C* (**H**), miR160 (**I**), *eTM160-1* (**J**), *eTM160-2* (**K**), *ARF10* (**L**), *ARF16* (**M**), *ARF17* (**N**), *DRB1* (**O**), *DRB2* (**P**), and *DRB4* (**Q**). (**B**–**Q**) All phenotypic and molecular analyses relied on the use of three biological replicates with each biological replicate consisting of a pool of six plants. Altered development (**B**–**E**) or a change in transcript abundance (**F**–**Q**) was determined via comparison of the *drb1*/0.1 μM and *drb1*/1.0 μM treated samples to the values obtained for the untreated *drb1*/0.0 μM control sample by a standard two-tailed *t*-test. Error bars represent the standard error of the mean (SEM) and an asterisk (*) shows * *p* ≤ 0.05, ** *p* ≤ 0.01, *** *p* ≤ 0.001.

**Figure 3:**
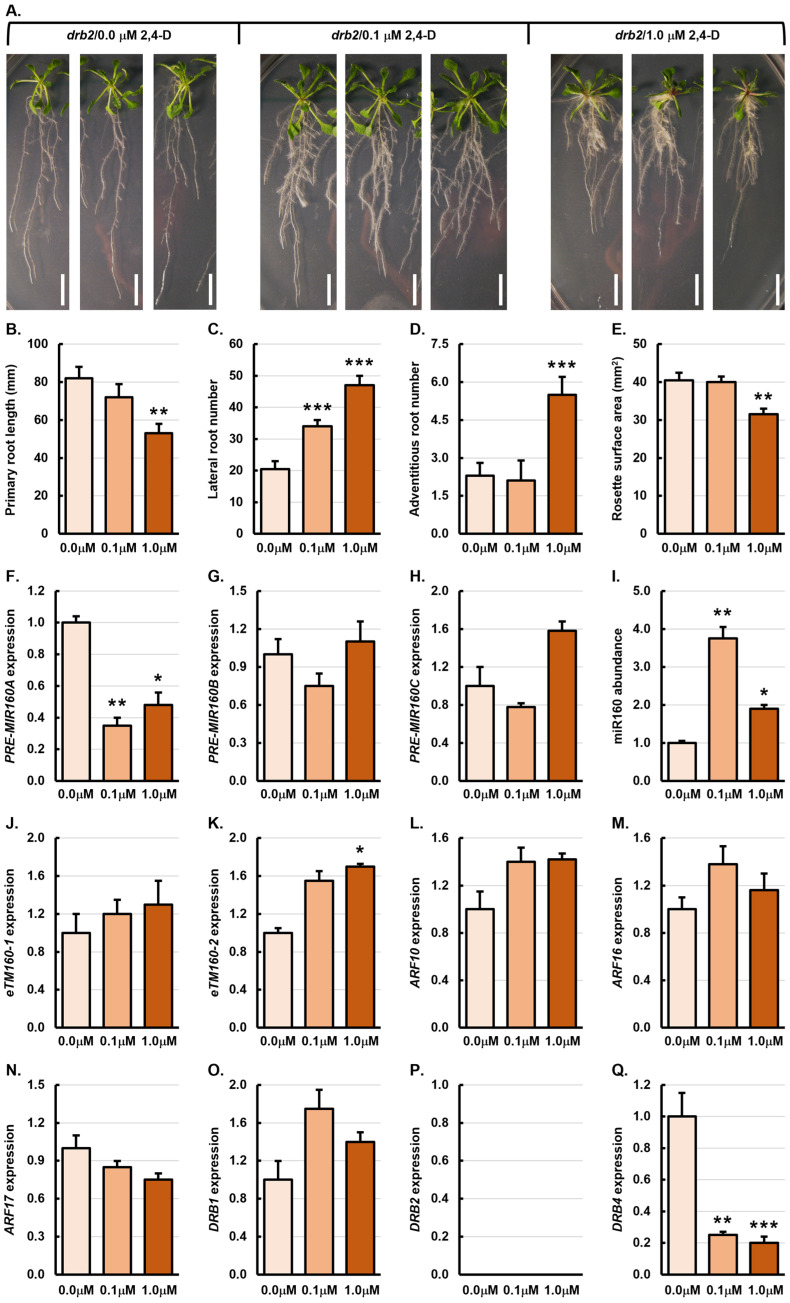
Phenotypic and molecular analysis of 21-day-old *drb2* plants treated with 2,4-D. (**A**) Representative phenotypes displayed by 21-day-old *drb2* control plants or following their treatment with 0.1 μM and 1.0 μM 2,4-D. Scale bar = 1.0 cm. (**B**–**E**) Quantification of primary root length (**B**), lateral root number (**C**), adventitious root number (**D**), and rosette leaf surface area (**E**) of 21-day-old *drb2* control plants or following treatment of *drb2* plants with 0.1 μM and 1.0 μM 2,4-D. (**F**–**Q**) Molecular analysis of all components of the miR160 expression module in the roots of *drb2*/0.0 μM, *drb2*/0.1 μM and *drb2*/1.0 μM plants, including assessment of the expression of *PRE-MIR160A* (**F**), *PRE-MIR160B* (**G**), *PRE-MIR160C* (**H**), miR160 (**I**), *eTM160-1* (**J**), *eTM160-2* (**K**), *ARF10* (**L**), *ARF16* (**M**), *ARF17* (**N**), *DRB1* (**O**), *DRB2* (**P**), and *DRB4* (**Q**). (**B**–**Q**) All phenotypic and molecular analyses relied on the use of three biological replicates and each replicate consisted of a pool of six plants. Altered development (**B**–**E**) or a change in transcript abundance (**F**–**Q**) was determined via comparison of the *drb2*/0.1 μM and *drb2*/1.0 μM treated samples to the values obtained for the *drb2*/0.0 μM control sample by a standard two-tailed *t*-test. Error bars represent the standard error of the mean (SEM) and an asterisk (*) shows * *p* ≤ 0.05, ** *p* ≤ 0.01, *** *p* ≤ 0.001.

**Figure 4:**
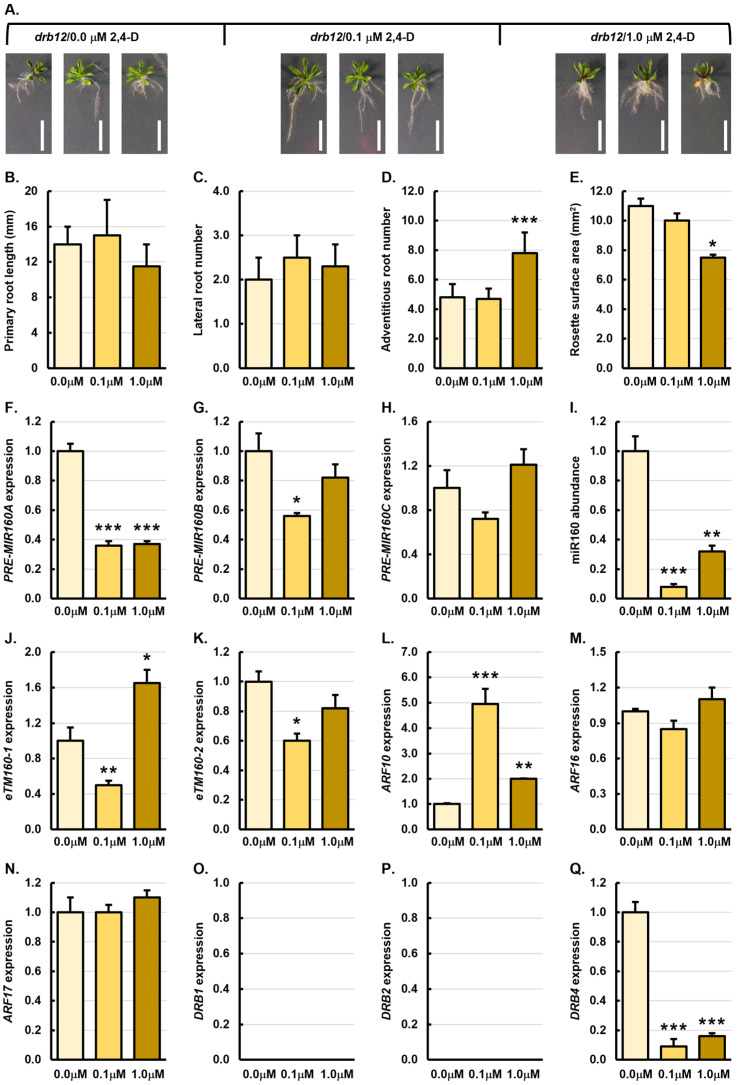
Phenotypic and molecular analysis of 21-day-old *drb12* plants treated with 2,4-D. (**A**) Phenotypes displayed by 21-day-old *drb12* control plants or following their treatment with 0.1 μM and 1.0 μM 2,4-D. Scale bar = 1.0 cm. (**B**–**E**) Quantification of primary root length (**B**), lateral root number (**C**), adventitious root number (**D**), and rosette leaf surface area (**E**) of 21-day-old *drb12*/0.0 μM, *drb12*/0.1 μM and *drb12*/μM plants. (**F**–**Q**) Molecular analysis of all miR160 expression module components in the roots of 21-day-old *drb12*/0.0 μM, *drb12*/0.1 μM and *drb12*/μM plants, including assessing *PRE-MIR160A* (**F**), *PRE-MIR160B* (**G**), *PRE-MIR160C* (**H**), miR160 (**I**), *eTM160-1* (**J**), *eTM160-2* (**K**), *ARF10* (**L**), *ARF16* (**M**), *ARF17* (**N**), *DRB1* (**O**), *DRB2* (**P**), and *DRB4* (**Q**) expression. (**B**–**Q**) All phenotypic and molecular analyses relied on the use of three biological replicates and each replicate consisted of a pool of six plants. Altered development (**B**–**E**) or a change in transcript abundance (**F**–**Q**) was determined via comparison of the *drb12*/0.1 μM and *drb12*/1.0 μM samples to the values obtained for the *drb12*/0.0 μM control sample by a standard two-tailed *t*-test. Error bars represent the standard error of the mean (SEM) and an asterisk (*) shows * *p* ≤ 0.05, ** *p* ≤ 0.01, *** *p* ≤ 0.001.

**Figure 5:**
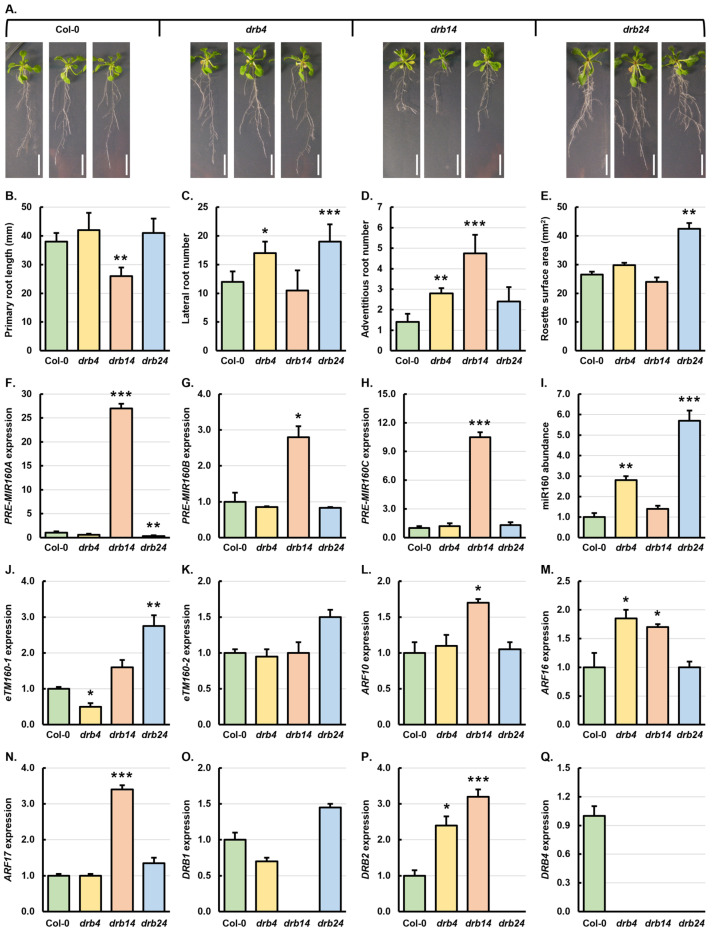
Molecular characterization of the miR160 expression module in the roots of 21-day-old Col-0, *drb4*, *drb14*, and *drb24* plants. (**A**) Representative phenotypes displayed by 21-day-old Col-0, *drb4*, *drb14*, and *drb24* plants. Scale bar = 1.0 cm. (**B**–**E**) Quantification of primary root length (**B**), lateral root number (**C**), adventitious root number (**D**), and rosette leaf surface area (**E**) of 21-day-old Col-0, *drb4*, *drb14*, and *drb24* plants. (**F**–**Q**) Molecular analysis of all components of the miR160 expression module in the roots of 21-day-old Col-0, *drb4*, *drb14*, and *drb24* plants, including profiling the expression of *PRE-MIR160A* (**F**), *PRE-MIR160B* (**G**), *PRE-MIR160C* (**H**), miR160 (**I**), *eTM160-1* (**J**), *eTM160-2* (**K**), *ARF10* (**L**), *ARF16* (**M**), *ARF17* (**N**), *DRB1* (**O**), *DRB2* (**P**), and *DRB4* (**Q**). (**B**–**Q**) All phenotypic and molecular analyses relied on the use of three biological replicates with each replicate consisting of a pool of six plants. Altered development (**B**–**E**) or a change in transcript abundance (**F**–**Q**) was determined via comparison of the *drb4*, *drb14*, and *drb24* samples to the values obtained for Col-0 plants by a standard two-tailed *t*-test. Error bars represent the standard error of the mean (SEM) and an asterisk (*) shows * *p* ≤ 0.05, ** *p* ≤ 0.01, *** *p* ≤ 0.001.

The data generated for statistical analysis in this study were obtained from three biological replicates of each analyzed plant line and each biological replicate consisted of a pool of six individual 21-day-old plants. Statistical analysis was performed using a standard two-tailed *t*-test. Different degrees of statistical significance are represented in Figure 1, Figure 2, Figure 3, Figure 4 and Figure 5 via the use of an asterisk (*), where * *p* ≤ 0.05, ** *p* ≤ 0.01, and *** *p* ≤ 0.001. The absence of an asterisk above a column in the graphs presented in Figure 1, Figure 2, Figure 3 and Figure 4 indicates that there was no statistically significance difference between the control sample (0.0 μM 2,4-D) and 0.1 and 1.0 μM 2,4-D treated samples. Similarly, in Figure 5, the lack of an asterisk above a column in the presented graphs shows no statistically significant difference between Col-0 plants and the *drb4*, *drb14*, and *drb24* mutants.

## 3. Results

### 3.1. Phenotypic and Molecular Analysis of Wild-Type Arabidopsis Plants Treated with the Synthetic Auxin 2,4-Dichlorophenoxyacetic Acid

The miR160/*ARF10*/*ARF16*/*ARF17* expression module provides an excellent system to study the complex interaction between miRNA-mediated regulation of *ARF* gene expression and *Arabidopsis* root development. Two concentrations of the synthetic auxin 2,4-dichlorophenoxyacetic acid (2,4-D) were applied to wild-type *Arabidopsis* (Col-0) plants, and to the *drb1*, *drb2*, and *drb12* mutants via direct root contact. The period of exposure to 2,4-D was limited to 24 h, and the phenotypic changes and molecular alterations stemming from treatment with the two assessed synthetic auxin concentrations in Col-0, *drb1*, *drb2*, and *drb12* plants were observed 10 days later to uncover the relationship between modification of the auxin signaling pathway and miR160-directed regulation of *ARF10*, *ARF16*, and *ARF17* expression.

Figure 1A shows that at the whole plant level, the application of 1.0 μM 2,4-D impacted the development of Col-0 seedlings to a much greater degree than 0.1 μM 2,4-D and, considering that primary root growth is one of the major developmental processes regulated by auxin [40], this formed the first phenotypic metric assessed. The average length of the primary root of a 21-day-old Col-0 seedling was 38 millimeters (mm) (Figure 1B). The primary root length of 0.1 μM treated Col-0 (Col-0/0.1 μM) seedlings was significantly elevated by 18.4% to 45 mm and elevated to a lesser degree (10.5%) in 1.0 μM Col-0 (Col-0/1.0 μM) plants to 42 mm (Figure 1B). Lateral and adventitious root development has also been shown to be influenced by exogenous auxin application [39,41,42,43], or via molecular modification of components of the miR160 expression module [12,39]; therefore, these two phenotypic metrics were also quantified. Untreated 21-day-old Col-0 plants developed an average of 12 lateral and 1.5 adventitious roots (Figure 1C,D). In 0.1 μM and 1.0 μM 2,4-D treated Col-0 plants, lateral root development was significantly promoted by 83.3% and 200.8% with an average of 22 and 37 lateral roots forming in Col-0/0.1 μM and Col-0/1.0 μM plants, respectively (Figure 1C). In Col-0/0.1 μM plants, adventitious root development was only mildly enhanced by 20.0% with an average of 1.8 adventitious roots forming per plant (Figure 1D). However, adventitious root development was significantly enhanced by 193.3% in Col-0/1.0 μM plants with an average of 4.4 adventitious roots forming after treatment with the higher concentration of 2,4-D (Figure 1D). The phenotypic properties displayed by the rosette and other aerial tissues of an *Arabidopsis* plant also form excellent indicators of overall plant health, and the communication status between root and shoot tissues in response to the external environment [44,45,46]. The total surface area of the rosette of Col-0 plants treated with 0.1 μM 2,4-D was only mildly increased by 3.7% to 28 mm squared (mm^2^) from an average rosette surface area of 27 mm^2^ for Col-0/0.0 μM plants (Figure 1E). In contrast, the application of 1.0 μM 2,4-D had a negative impact on Col-0 aerial tissue development, with the total surface area of the rosette of Col-0/1.0 μM plants significantly reduced by 24.7% to 20.5 mm^2^ (Figure 1E).

At the molecular level, the miR160 expression module forms a highly complex module in *Arabidopsis* with the miR160 sRNA processed from three precursor transcripts transcribed from three *MIR* genes at distinct genome positions, specifically loci *MIR160A* (*AT2G39175*), *MIR160B* (*AT4G17788*), and *MIR160C* (*AT5G46845*). Furthermore, post its processing from the *PRE-MIR160A*, *PRE-MIR160B*, and *PRE-MIR160C* precursors via either DRB1-dependent or DRB2-dependent mechanisms [25,28,39], the abundance of miR160 is further regulated in a spatiotemporal manner by two endogenous target mimics (*eTM*s), *eTM160-1* and *eTM160-2* [47,48], which together, fine tune the degree of miR160-directed regulation of *ARF10*, *ARF16*, and *ARF17* gene expression [12,13,39].

Compared to the roots of Col-0/0.0 μM plants, *PRE-MIR160A*, *PRI-MIR160B*, and *PRE-MIR160C* transcript abundance was reduced by 1.8-, 1.3-, and 2.0-fold in the roots of Col-0/0.1 μM plants (Figure 1F–H). In Col-0/1.0 μM roots, *PRE-MIR160A* transcript abundance remained unchanged (Figure 1F), while the level of expression of *PRE-MIR160B* and *PRE-MIR160C* was elevated by 1.9-fold (Figure 1G) and reduced by 1.4-fold, respectively (Figure 1H). Decreased precursor transcript abundance in Col-0/0.1 μM roots suggested that these three precursors were more efficiently processed following treatment of Col-0 plants with 0.1 μM 2,4-D, which was in turn, expected to result in higher levels of miR160 accumulation. However, the abundance of miR160 was greatly reduced by 10.0-fold in Col-0/0.1 μM roots (Figure 1I), and not elevated as expected. Considering that *PRE-MIR160A* abundance remained unchanged, *PRE-MIR160B* levels were significantly elevated, and that *PRE-MIR160C* abundance was moderately reduced in Col-0 roots following treatment with 1.0 μM 2,4-D (Figure 1F–H), a more tempered alteration to miR160 abundance was expected. Accordingly, miR160 levels were reduced by 2.0-fold in Col-0/1.0 μM roots (Figure 1I). RT-qPCR next revealed that *eTM160-1* and *eTM160-2* transcript abundance was mildly decreased by 1.3-fold and 1.1-fold, respectively (Figure 1J,K), in response to the significant decrease in the level of their regulated miR160 in Col-0/0.1 μM roots (Figure 1I). In contrast, the abundance of the *eTM160-1* transcript was significantly elevated by 6.8-fold (Figure 1J), and the level of *eTM160-2* remained unchanged in Col-0/1.0 μM roots (Figure 1K), compared to their levels in Col-0/0.0 μM roots.

In response to significantly reduced miR160 accumulation in Col-0/0.1 μM roots, *ARF10* expression was mildly reduced by 1.1-fold (Figure 1L), *ARF16* expression was significantly elevated by 5.0-fold (Figure 1M), and *ARF17* expression was mildly increased by 1.1-fold (Figure 1N). Taken together, these target gene expression trends indicated that only the *ARF16* target gene was responsive to altered miR160 abundance in 0.1 μM 2,4-D treated Col-0 roots. In Col-0/1.0 μM roots where miR160 accumulation was reduced by 2.0-fold (Figure 1I), *ARF10* and *ARF17* gene expression was also mildly reduced by 1.1- and 1.3-fold, respectively (Figure 1L,N), and *ARF16* expression was elevated by 1.4-fold (Figure 1M). Again, these target gene expression trends indicated that of the three miR160 targets assessed, only *ARF16* was responsive to altered miR160 accumulation in the roots of Col-0 plants after treatment with exogenous auxin. To attempt to provide further insight into the transcript abundance trends documented for some components of the miR160 expression module in the roots of 21-day-old Col-0 plants treated with 2,4-D, the level of expression of the three nucleus-localized DRB proteins assigned functional roles in miRNA production in *Arabidopsis*, including *DRB1*, *DRB2*, and *DRB4*, was also assessed via RT-qPCR. In Col-0/0.1 μM roots, *DRB1*, *DRB2* and *DRB4* expression was reduced by 1.2-, 1.1-, and 1.6-fold, respectively (Figure 1O–Q). In Col-0/1.0 μM roots, *DRB1* expression was mildly increased by 1.3-fold (Figure 1O), and the expression of *DRB2* and *DRB4* was significantly reduced by 2.2- and 1.6-fold, respectively (Figure 1P,Q).

Molecular profiling of components at each stage of the miR160 expression module failed to identify expected abundance trends for a number of the components assessed via RT-qPCR in Col-0 roots following their treatment with 2,4-D. Taking the findings made in Col-0/0.1 μM roots as an example, the transcript abundance of *PRE-MIR160A*, *PRE-MIR160B*, *PRE-MIR160C*, miR160, *eTM160-1*, *eTM160-2*, *ARF10*, and *ARF17* were all reduced. However, reduced precursor transcript abundance was expected to result in elevated miR160 abundance due to enhanced precursor transcript processing efficiency, and similarly, reduced miR160 abundance was expected to increase *ARF* target gene expression due to relaxation of miR160-directed expression regulation. However, the RT-qPCR analyses presented in Figure 1 did not identify these expected transcript abundance trends. Therefore, we next applied a bioinformatic approach to profile the promoter landscapes of the encoding loci of the individual components of the miR160 expression module, which were assessed by RT-qPCR in Col-0 roots to obtain a more thorough understanding of the influence which exogenous auxin application may have had on the transcriptional activity of each analyzed component of the miR160 expression module. The nucleotide sequences of known *ARE*s in *Arabidopsis* gene promoters, including *TGTCTC*, *GAGACA*, *AGAAACAT*, *AGAAACAA*, and *NGATT* (where ‘*N*’ represents any nucleotide) were aligned to the putative promoter regions (two kilobases (2 kb) of the genomic DNA sequence immediately upstream of the transcription start site of each gene) of each gene encoding a component of the miR160 expression module using three different online programs, including PlantCARE [49], PLACE [50], and AtcisDB [51].

**Table 1:** Bioinformatic assessment of the *cis*-acting element landscape of the promoter regions of miR160 expression module genes to determine the presence of *ARE*s.

Gene Name	PlantCARE [49]	PLACE [50]	AtcisDB [51]
*MIR160A*	**×**	✔	N/A
*MIR160B*	✔	✔	N/A
*MIR160C*	✔	**×**	N/A
*eTM160-1*	**×**	✔	N/A
*eTM160-2*	**×**	✔	N/A
*ARF10*	✔	✔	✔
*ARF16*	**×**	✔	✔
*ARF17*	✔	**×**	✔
*DRB1*	**×**	✔	**×**
*DRB2*	✔	**×**	**×**
*DRB4*	**×**	✔	✔

Confirmation of the presence of an *ARE* in the promoter region of a miR160 expression module gene is denoted by the check mark (✔) symbol, whereas the failure to identify an *ARE* in a promoter region of a miR160 expression module gene by the three online programs is denoted by a cross (**×**) symbol.

Three different programs were used for this analysis as each program uses a unique set of parameters to determine the presence or absence of *cis*-acting elements in gene promoters. Therefore, utilizing this program combination was considered the best strategy to obtain a comprehensive overview of the *ARE cis*-acting element landscape of each miR160 expression module gene. Table 1 shows that *ARF10* was the only gene identified by all three programs to harbor an *ARE* in its promoter region. Loci *MIR160B*, *ARF16*, and *ARF17*, as well as *DRB4*, which was included in this analysis due to its known role in tasiARF production [52], were identified by two of the three programs to harbor an *ARE*(s) in their promoter regions. A higher level of confidence in *ARE* identification in the promoter regions of the 11 assessed genes could not be achieved via the applied bioinformatic approach as the AtcisDB program is not capable of assessing the promoter region *cis*-acting element landscapes of non-coding genes in addition to its ability to interrogate the *cis*-acting element landscapes of protein-coding gene promoters. However, at least one of the three programs used did confirm the presence of an *ARE* in the promoter regions of the six remaining miR160 module genes also included in this in silico analysis, including *ARE* identification in the promoter regions of the *MIR160A*, *MIR160C*, *ETM160-1*, *ETM160-2*, *DRB1*, and *DRB2* genes (Table 1). When the Table 1 data are considered together with the transcript abundance trends presented in Figure 1, these findings (1) identify all analyzed miR160 expression module genes as *ARG*s due to the presence of *ARE cis*-acting elements in their promoter regions, and (2) suggest that at least some of the unexpected abundance trends identified by RT-qPCR are the result of the influence of the exogenously applied 2,4-D at the transcriptional level, in addition to the multilayered regulatory complexity of various components of the miR160 expression module at the posttranscriptional level.

### 3.2. Phenotypic and Molecular Analysis of the drb1 Single Mutant Treated with the Synthetic Auxin 2,4-Dichlorophenoxyacetic Acid

Figure 2A shows the severely impeded development of the *drb1* mutant, a phenotype which has been described in detail previously [22,28,38] and which is the result of defective production of most *Arabidopsis* miRNAs in the absence of DRB1 forming a functional partnership with DCL1 [23,24,25,26,27,28]. Figure 2A also readily shows that treatment of *drb1* plants with 0.1 μM 2,4-D (*drb1*/0.1 μM plants) enhanced the development of both aerial structures and the root system of *drb1*/0.1 μM plants, while treatment of *drb1* plants with 1.0 μM 2,4-D (*drb1*/1.0 μM plants) appeared to only impact primary root development. Promotion of phenotypic properties of the aerial and root tissues of *drb1* plants following treatment with 0.1 μM 2,4-D was confirmed via quantification of primary root length, lateral and adventitious root number, and rosette surface area (Figure 2B–E). More specifically, the primary root length of *drb1*/0.1 μM plants was significantly increased by 46.2% to 38 mm from 26 mm for untreated *drb1* control plants (*drb1*/0.0 μM plants) (Figure 2B). Similarly, lateral root formation was also significantly enhanced in *drb1*/0.1 μM plants, increased by 177.1% with *drb1*/0.1 μM plants forming an average of 9.7 lateral roots, compared to the average lateral root number of 3.5 for *drb1*/0.0 μM plants (Figure 2C). Although not to the same degree as determined for primary root length and lateral root number in *drb1*/0.1 μM plants, adventitious root number and rosette surface area were also enhanced in *drb1* mutant plants treated with the lower concentration of 2,4-D (Figure 2D,E). Moreover, the *drb1* control plants formed an average of 4.1 adventitious roots and developed a rosette with an average surface area of 16.5 mm^2^, whereas the adventitious root number was increased by 17.1% to 4.8 (Figure 2D), and the rosette surface area was increased by 19.4% to 19.7 mm^2^ (Figure 2E) in *drb1*/0.1 μM plants. In direct contrast to the promotion of primary root length in *drb1*/0.1 μM plants, primary root length was significantly reduced by 32.7% to 17.5 mm in *drb1*/1.0 μM plants (Figure 2B). The phenotypic metrics of the lateral root number, adventitious root number, and rosette surface area all showed mild alterations in *drb1*/1.0 μM plants compared to *drb1*/0.0 μM plants. Namely, lateral and adventitious root numbers were increased by 14.3% and 12.2% to 4.0 and 4.6 lateral and adventitious roots, respectively (Figure 2C,D), and rosette surface area was reduced by 6.1% to 15.5 mm^2^ (Figure 2E).

The level of *PRE-MIR160A*, *PRE-MIR160B*, and *PRE-MIR160C* expression was significantly reduced by 2.2-, 2.0-, and 2.2-fold, respectively, in *drb1*/0.1 μM roots compared to the level of expression of the three miR160 precursor transcripts in *drb1*/0.0 μM roots (Figure 2F–H). Accordingly, miR160 abundance was found to be significantly elevated by 5.0-fold in *drb1*/0.1 μM roots compared to its level in *drb1*/0.0 μM roots (Figure 2I), a finding which indicated that elevated miR160 abundance was the result of enhanced precursor transcript processing efficiency in the roots of the *drb1* mutant after its treatment with 0.1 μM 2,4-D (Figure 2F–I). The level of both *eTM*s was also increased in *drb1*/0.1 μM roots, with *eTM160-1* levels increased by 2.8-fold (Figure 2J) and *eTM160-2* transcript abundance elevated by 1.2-fold (Figure 2K), expression trends that indicated that both *eTM*s were scaling in abundance according to the level of their regulated miRNA (Figure 2I). In response to elevated miR160 abundance in *drb1*/0.1 μM roots, *ARF10* expression was reduced by 1.8-fold (Figure 2L), and the expression of the *ARF16* and *ARF17* target genes was elevated by 1.9- and 1.1-fold, respectively (Figure 2M,N).

Accordingly, RT-qPCR failed to detect the expression of *DRB1* in the roots of *drb1*/0.1 μM plants (Figure 2O) but showed that the expression of *DRB2* was mildly reduced by 1.1-fold (Figure 2P) and that *DRB4* expression was significantly decreased by 2.4-fold in *drb1* roots following treatment with 0.1 μM 2,4-D (Figure 2Q). When considered together, the *DRB* expression trends suggested that, in the absence of *DRB1* expression, highly reduced *DRB4* expression, and therefore the removal of DRB4 protein antagonism of DRB2 protein function [34,37,38], likely allowed for enhanced miR160 precursor transcript efficiency via DRB2 forming a functional partnership with DCL1, leading to elevated miR160 abundance in *drb1*/0.1 μM roots.

In *drb1*/1.0 μM roots, compared to the root tissues of *drb1*/0.0 μM plants, *PRE-MIR160A*, *PRE-MIR160B*, and *PRE-MIR160C* expression was only mildly reduced by 1.3-, 1.3- and 1.1-fold, respectively (Figure 2F–H). Decreased miR160 precursor abundance in *drb1*/1.0 μM plants was again determined to be the result of enhanced precursor transcript processing efficiency, with miR160 accumulation elevated by 2.3-fold in the root tissues of *drb1* plants following their treatment with 1.0 μM 2,4-D. The abundance of *eTM160-1* and *eTM160-2* were elevated by 2.8- and 1.6-fold, respectively (Figure 2J,K). These *eTM* abundance trends suggested that (1) both transcripts were scaling in abundance along with that of their regulated miRNA, miR160, and (2) increased *eTM160* levels may have masked the actual degree of miR160 accumulation upregulation in *drb1*/1.0 μM roots. The *ARF* target gene expression trends documented in *drb1*/1.0 μM roots closely matched those obtained for the roots of *drb1*/0.1 μM plants (Figure 2L–N). Specifically, *ARF10* expression was reduced by 1.2-fold, and the expression of both *ARF16* and *ARF17* was elevated by 1.2-fold in *drb1*/1.0 μM roots. *DRB2* and *DRB4* expression trends in *drb1*/1.0 μM roots also closely aligned with those determined for *drb1*/0.1 μM roots, with *DRB2* and *DRB4* expression decreased by 2.0- and 1.6-fold in the root tissues of *drb1* plants treated with 1.0 μM 2,4-D (Figure 2P,Q). This result again suggested that, in the absence of DRB1 function, reduced *DRB4* expression and thereby the removal of DRB4 antagonism of DRB2 function, likely led to the enhanced efficiency of miR160 precursor transcript processing via the DRB2 protein forming a functional interaction with DCL1.

### 3.3. Phenotypic and Molecular Analysis of the drb2 Single Mutant Treated with the Synthetic Auxin 2,4-Dichlorophenoxyacetic Acid

Treatment of the *drb2* single mutant with either 0.1 or 1.0 μM 2,4-D had an inhibitory effect on the elongation of the primary root of *drb2*/0.1 μM and *drb2*/1.0 μM plants, while both 2,4-D concentrations promoted lateral root formation (Figure 3A). These observations were confirmed via quantification of the phenotypic metrics, primary root length, lateral and adventitious root number, and rosette surface area (Figure 2B–E). The average primary root length of untreated *drb2* control plants was 82 mm, with the average primary root length of *drb2*/0.1 μM and *drb2*/1.0 μM plants reduced by 12.2% and 35.4% to 72 mm and 53 mm, respectively (Figure 2B). The average number of lateral and adventitious roots formed by *drb2*/0.0 μM plants was 20.5 and 2.3, respectively (Figure 2C,D). In *drb2*/0.1 μM plants, the lateral root number was increased by 65.9% to 34 lateral roots per plant, and in *drb2*/1.0 μM plants, lateral root development was further promoted by 129.3% to an average of 47 lateral roots per plant (Figure 2C). Adventitious root formation was affected differently in the *drb2* mutant following its treatment with the two 2,4-D concentrations, with the adventitious root number mildly decreased by 9.1% to 2.1 adventitious roots per *drb2*/0.1 μM plant, and significantly increased by 139.1% to an average of 5.5 adventitious roots forming per *drb2*/1.0 μM plant (Figure 2D). The average rosette surface area for *drb2*/0.0 μM plants is 40.5 mm^2^, with the rosette surface area only mildly decreased by 1.3% to 40 mm^2^ in *drb2*/0.1 μM plants, whereas the rosette surface area of a *drb2*/1.0 μM plant was significantly decreased by 23.5% to 31.5 mm^2^ (Figure 2E).

Compared to their level of expression in *drb2*/0.0 μM roots, *PRE-MIR160A* was significantly reduced by 2.9-fold (Figure 3F), and the abundance of both *PRE-MIR160B* and *PRE-MIR160C* was mildly reduced by 1.3-fold in the roots of *drb2*/0.1 μM plants (Figure 3G,H). The accumulation level of miR160 was significantly increased by 3.8-fold in *drb2*/0.1 μM roots (Figure 3I), indicating that reduced precursor transcript abundance was the result of enhanced processing efficiency of the three miR160 precursors in the roots of *drb2* plants after the application of 0.1 μM 2,4-D. The level of the *PRE-MIR160A* transcript was also reduced in *drb2*/1.0 μM roots (down 2.1-fold). However, the level of the *PRE-MIR160B* and *PRE-MIR160C* transcripts was elevated by 1.1- and 1.6-fold, respectively, in *drb2* roots following treatment with 1.0 μM 2,4-D. Compared to its level of abundance in the roots of untreated *drb2* control plants, miR160 accumulation was elevated by 1.9-fold in *drb2*/1.0 μM roots (Figure 3I). This miRNA abundance trend was likely the result of increased efficiency in the processing of the *PRE-MIR160A* precursor (Figure 3F). In the roots of *drb2*/0.1 μM and *drb2*/1.0 μM plants, the abundance of both the *eTM160-1* and *eTM160-2* transcripts was increased to a similar degree (Figure 3J,K), a finding that once again indicated that the *eTM* transcripts were scaling in abundance in accordance to the level of their regulated miRNA.

In response to the 3.8-fold elevation in miR160 abundance in *drb2*/0.1 μM roots, *ARF10* and *ARF16* expression was increased by 1.4-fold, and *ARF17* expression was reduced by 1.2-fold (Figure 3L–N). A similar target gene expression trend was determined by RT-qPCR in *drb2*/1.0 μM roots, with *ARF10* and *ARF16* expression elevated by 1.4- and 1.2-fold, respectively (Figure 3L,M), and *ARF17* decreased by 1.3-fold (Figure 3N). The target gene expression trends for the roots of *drb2* plants treated with 0.1 and 1.0 μM 2,4-D suggest the either (1) only the *ARF17* target gene is responsive to the 2,4-D-induced alteration of miR160 abundance, or (2) translational repression forms the predominant mode of RNA silencing directed by miR160 to regulate the transcript abundance of its target genes, *ARF10* and *ARF16* (Figure 3I,L–N). Compared to *drb2*/0.0 μM roots, *DRB1* expression was elevated by 1.8- and 1.4-fold in the roots of *drb2*/0.1 μM and *drb2*/1.0 μM plants, respectively (Figure 3O), while the expression of *DRB4* was significantly reduced by 4.0- and 5.0-fold in these two 2,4-D treated samples (Figure 3Q). Elevated *DRB1* expression, and therefore increased DRB1 protein abundance, supports the suggestion that the processing efficiency of all three miR160 precursors was enhanced in *drb2*/0.1 μM roots (Figure 3F–H), as well as for the *PRE-MIR160A* precursor in *drb2*/1.0 μM roots (Figure 3F), resulting in the elevated abundance of miR160 in these two *drb2* samples following their treatment with 2,4-D (Figure 3I).

### 3.4. Phenotypic and Molecular Analysis of the drb12 Double Mutant Treated with the Synthetic Auxin 2,4-Dichlorophenoxyacetic Acid

Figure 4A shows that of the four *Arabidopsis* lines treated with 2,4-D, and when compared to untreated control plants (*drb12*/0.0 μM plants), the *drb12* double mutant appeared to be the least responsive to synthetic auxin treatment. The lack of response of *drb12* plants to 2,4-D treatment may in part stem from the already severely impeded development of this *Arabidopsis* line, a phenotypic consequence which results from loss of both DRB1 and DRB2 function as part of miRNA production [35,37,38]. The average primary root length of 21-day-old *drb12* control plants is 14 mm. In *drb12*/0.1 μM plants, the primary root length was increased by 7.1% to 15 mm, whereas the primary root length of *drb12*/1.0 μM plants was reduced by 17.9% to 11.5 mm (Figure 4B). The average number of lateral and adventitious roots of *drb12*/0.0 μM plants was 2.0 and 4.8 roots, respectively (Figure 4C,D). In *drb12*/0.1 μM plants, the lateral root number was promoted by 25% to 2.5 lateral roots, whereas the adventitious root number remained largely unchanged (down by 2.5%), with *drb12*/0.1 μM plants forming on average, 4.7 adventitious roots (Figure 4C,D). Compared to *drb12*/0.0 μM plants, both root phenotypic metrics were promoted in the *drb12* double mutant following its treatment with 1.0 μM 2,4-D, with *drb12*/1.0 μM plants developing an average of 2.3 and 7.8 lateral and adventitious roots, representing 15.0% and 62.5% increases, respectively (Figure 4C,D). Aerial tissue development was impeded in both the *drb12*/0.1 μM and *drb12*/1.0 μM samples, compared to the rosette surface area of 11 mm^2^ for *drb12*/0.0 μM plants. Specifically, the average rosette surface area of *drb12*/0.1 μM plants was reduced by 9.1% to 10 mm^2^ and the average rosette surface area of *drb12*/1.0 μM plants was reduced by 31.9% to 7.5 mm^2^ (Figure 4E).

The abundance of *PRE-MIR160A*, *PRE-MIR160B*, and *PRE-MIR160C* was reduced by 2.8-, 1.8-, and 1.4-fold, respectively, in *drb12*/0.1 μM roots, compared to *drb12*/0.0 μM roots (Figure 4F–H). The accumulation level of miR160 was significantly reduced by 12.5-fold in *drb12*/0.1 μM roots (Figure 4I). This accumulation trend indicated that reduced miR160 precursor transcript abundance was the result of decreased *MIR160A*, *MIR160B*, and *MIR160C* gene activity in *drb12*/0.1 μM roots after synthetic auxin treatment (Figure 4F–I). The abundance of the *PRE-MIR160A* and *PRE-MIR160B* transcripts were also reduced by 2.7- and 1.2-fold in the roots of the *drb12* double mutant following its treatment with 1.0 μM 2,4-D (Figure 4F,G). However, *PRE-MIR160C* abundance was mildly elevated by 1.2-fold in *drb12*/1.0 μM roots (Figure 4H). Considering that miR160 abundance was significantly reduced by 3.1-fold in *drb12*/1.0 μM roots, the expression trends documented for *PRE-MIR160A* and *PRE-MIR160B* again suggest that the transcriptional activity of the *MIR160A* and *MIR160B* loci are repressed by 2,4-D application. Furthermore, a decreased level of miR160 accumulation in *drb12*/1.0 μM roots additionally indicated that elevated *PRE-MIR160C* abundance could not compensate for the reduced levels of the *PRE-MIR160A* and *PRE-MIR160B* precursors in the absence of DRB1 and DRB2 function. The abundance of the *eTM160-1* and *eTM160-2* transcripts was decreased by 2.0- and 1.7-fold in *drb12*/0.1 μM roots, indicating that the levels of both *eTM*s were scaling in abundance according to the level of their regulated miRNA, miR160 (Figure J,K). The level of *eTM106-2* was also reduced in the roots of *drb12* plants following treatment with 1.0 μM 2,4-D (Figure 4K). This once again indicated that the level of this miR160-regulating *eTM* was scaling in accordance with the level of abundance of miR160 (Figure 4I). In contrast, *eTM160-1* levels were increased by 1.7-fold in *drb12*/1.0 μM roots (Figure 4J), a finding which suggested that the regulatory ability of *eTM160-1* to respond to altered miR160 abundance became defective in the roots of *drb12* plants following their treatment with the higher concentration of 2,4-D. Alternatively, this finding could indicate that the transcriptional activity of the *eTM160-1* encoding locus was promoted by the application of the higher 2,4-D concentration.

The expression level of *ARF10* was significantly elevated by 5.0-fold in *drb12*/0.1 μM roots and by 2.0-fold in *drb12*/1.0 μM roots (Figure 4L). The *ARF10* expression trend indicated that miR160-directed regulation of *ARF10* expression was released in the roots of the *drb12* double mutant following its treatment with 2,4-D. In *drb12*/0.1 μM roots, the level of expression of *ARF16* and *ARF17* was mildly elevated by 1.2-fold and remained unchanged, respectively (Figure 4M,N). Similarly, the expression of both *ARF16* and *ARF17* was only mildly elevated by 1.1-fold in *drb12*/1.0 μM roots (Figure 4M,N). When the *ARF16* and *ARF17* expression trends are considered together with the level of miR160 accumulation in the roots of *drb12*/0.1 μM and *drb12*/1.0 μM plants, the transcript abundance trends indicated that, in the absence of DRB1 and DRB2 function, the *ARF16* and *ARF17* target transcripts lose their ability to appropriately respond to the level of their regulating miRNA, miR160 (Figure 4I,M,N). As expected, RT-qPCR failed to detect the expression of *DRB1* and *DRB2* in the roots of the *drb12* double mutant regardless of the 2,4-D treatment status of this *Arabidopsis* line (Figure 4O,P). Therefore, considering that in the roots of *drb12* plants, neither the DRB1 nor the DRB2 protein is present to antagonize the function of the DRB4 protein, it was unexpected to see the level of *DRB4* gene activity reduced to such a significant degree, down by 11.1- and 6.3-fold in *drb12*/0.1 μM and *drb12*/1.0 μM roots, respectively (Figure 4Q). However, this highly reduced level of *DRB4* expression, and therefore DRB4 protein abundance, may to a small degree, contribute to the considerable reduction to the level of miR160 accumulation in the roots of *drb12* plants after treatment with 2,4-D. This result also potentially uncovered a previously unknown role for DRB4 to provide an additional layer of regulatory complexity to the miR160 expression module.

### 3.5. Phenotypic and Molecular Analysis of the drb4, drb14, and drb24 to Establish a Regulatory Role for DRB4 in the miR160 Expression Module

Although the involvement of DRB4 in sRNA production in *Arabidopsis* has been shown to be restricted to siRNA production [34,38], including tasiARF production [52,53], and the production of the non-conserved subclass of miRNAs [28,33,34,38], the expression of *DRB4* was repressed to differing degrees in the roots of 21-day-old Col-0 (Figure 1Q), *drb1* (Figure 2Q), *drb2* (Figure 3Q), and *drb12* (Figure 4Q) seedlings following the treatment of these four *Arabidopsis* lines with 0.1 μM and 1.0 μM 2,4-D. Therefore, the miR160 expression module was next molecularly profiled in the roots of the *drb4* single mutant and the *drb14* and *drb24* double mutants to determine the contribution of DRB4 in the regulation of the miR160 expression module. Compared to the primary root length of Col-0 plants (38 mm), the primary root length of *drb4* plants was increased by 10.5% to 42 mm, decreased by 31.6% to 26 mm in *drb14* plants, and elevated by 7.9% to 41 mm in the *drb24* double mutant (Figure 5B). The average number of lateral roots for 21-day-old Col-0 plants was 12, with lateral root development significantly promoted by 41.7% (*n* = 17) and 58.3% (*n* = 19) in the *drb4* and *drb24* mutant backgrounds, respectively (Figure 5C). In contrast, lateral root development was mildly repressed by 12.5%, with *drb14* plants forming an average of 10.5 lateral roots. Adventitious root formation was promoted 100.0%, 239.2%, and 71.4%, with *drb4*, *drb14*, and *drb24* plants developing an average of 2.8, 4.8, and 2.4 adventitious roots, respectively, compared to Col-0 plants, which formed an average of 1.4 adventitious roots (Figure 5D). The rosette surface area of 21-day-old Col-0 plants was 26.5 mm^2^ (Figure 5E). In contrast, the rosette surface area of *drb4* and *drb24* plants was increased by 12.5% and 60.4% to 29.8 and 42.5 mm^2^, respectively, and reduced by 9.4% to 24.0 mm^2^ in the *drb14* double mutant (Figure 5E). Quantification of the phenotypic metrics displayed by the *drb14* and *drb24* double mutants (Figure 5B–E), and comparison of these to those quantified for the *drb1* (Figure 2B–E) and *drb2* single mutants (Figure 3B–E) revealed that the *drb4* phenotype was additive to the more developmentally severe phenotypes displayed by *drb1* and *drb2* plants.

The level of *PRE-MIR160A* and *PRE-MIR160C* transcript abundance was significantly elevated by 27.0- and 10.5-fold in *drb14* roots, respectively (Figure 5F,H), indicating that, in the absence of DRB1 and DRB4 function, miR160 production from these two miR160 precursor transcripts is largely defective. By comparison, *PRE-MIR160B* abundance was only mildly elevated by 2.8-fold in the roots of the *drb14* double mutant (Figure 5G), a transcript abundance trend which suggested that miR160 processing from this precursor remains somewhat functional in *drb14* roots, likely via the action of DRB2. In *drb4* and *drb24* roots, *PRE-MIR160A* levels were reduced by 1.7-fold and 3.3-fold, respectively (Figure 5F), suggesting that both DRB4 and DRB2 are antagonistic to DRB1 function in miR160 processing from this precursor. The *PRE-MIR160B* precursor was mildly reduced in abundance by 1.2-fold in *drb4* and *drb24* roots, and the *PRE-MIR160C* precursor was elevated by a similar degree (1.2 to 1.3-fold) in the root systems of these two *drb* mutant backgrounds when compared to Col-0 roots (Figure 5G,H). These transcript abundance trends again identify secondary roles for DRB2 and DRB4 in regulating the rate of miR160 production from these two precursors. In *drb4* and *drb24* roots, miR160 levels were significantly elevated by 2.8- and 5.7-fold, respectively (Figure 5I). When considered together with *PRE-MIR160* transcript abundance, the observed increase in miR160 levels in *drb4* and *drb24* roots (Figure 5I) most likely resulted from enhanced processing efficiency of *PRE-MIR160A* and *PRE-MIR160B* by the DRB1/DCL1 partnership (Figure 5F,G), in the absence of DRB2 or DRB4 antagonism of DRB1 function. Considering that *PRE-MIR160A*, *PRE-MIR160B*, and *PRE-MIR160C* levels were all significantly elevated in *drb14* roots, which suggested highly inefficient or even completely defective precursor transcript processing, it was unexpected that miR160 accumulation was elevated by 1.4-fold, and not reduced. However, this unexpected result does indicate that DRB2 can, to a minor degree, compensate for the loss of DRB1 function (and/or DRB4 function), to ensure that a reduced level of miR160 continues to accumulate in the roots of this double mutant plant.

In *drb4* roots, compared to Col-0 roots, *eTM160-1* abundance was reduced by 2.0-fold (Figure 5J). In contrast, *eTM160-1* levels were elevated by 1.6- and 2.8-fold in *drb14* and *drb24* roots, respectively. The level of *eTM160-2* did not differ greatly from its wild-type levels in the roots of *drb4*, *drb14*, and *drb24* plants. Moreover, *eTM160-2* levels were mildly reduced by 1.1-fold in *drb4* roots, unchanged in *drb14* roots, and elevated by 1.5-fold in *drb24* roots (Figure 5K). In response to elevated miR160 accumulation in *drb4*, *drb14*, and *drb24* roots (Figure 5I), *ARF10* expression was also elevated by 1.1-, 1.7-, and 1.1-fold in the roots of the three assessed *drb* mutants (Figure 5L). The expression of *ARF16* only returned an expected expression trend in *drb14* roots, with *ARF16* expression reduced by 1.7-fold (Figure 5M) in response to the 1.4-fold increase in miR160 levels (Figure 5I). In contrast, *ARF16* expression was elevated by 1.8-fold in *drb4* roots and remained unchanged from its wild-type level of expression in the roots of *drb24* plants (Figure 5M). The level of *ARF17* expression remained at wild-type levels in *drb4* roots and was elevated by 3.4- and 1.4-fold in the roots of *drb14* and *drb24* plants, respectively (Figure 5N). When considered together, the lack of transcript abundance response of the miR160 target genes *ARF10*, *ARF16*, and *ARF17* (Figure 5L–N), with respect to the level of alteration to miR160 accumulation (Figure 5I), indicated that, in the absence of *DRB4* gene expression (Figure 5Q), and hence when DRB4 protein function is defective, the ability of miR160 to appropriately regulate the expression of its three *ARF* target genes is compromised. Furthermore, the *ARF* target gene expression trends, like the miR160 precursor transcript abundance trends in *drb4*, *drb14*, and *drb24* roots (Figure 5F–H), adds further weight to our proposal that DRB4 does indeed play an important role in adding to the regulatory complexity of the miR160 expression module in *Arabidopsis* roots. The altered expression of *DRB1* in *drb4* and *drb24* roots (Figure 5O), and of *DRB2* in the roots of *drb4* and *drb14* plants (Figure 5P), also likely contributed to the molecular defects documented by RT-qPCR; these defects would have rendered miR160-directed regulation of *ARF10*, *ARF16*, and *ARF17* expression largely defective in the roots of *Arabidopsis* lines harboring knockout mutations in the loci that encode the DRB1, DRB2, and DRB4 proteins.

## 4. Discussion

### 4.1. Synthetic Auxin Application Altered Col-0 Root Development Due to Molecular Alteration of the miR160 Expression Module

As for most *Arabidopsis* miRNAs [22,23,24,25], the DRB1/DCL1 functional partnership acts as the primary mediator of miR160 production in *Arabidopsis* [25]. However, we have recently shown that in *Arabidopsis* roots, DRB2 also plays an essential functional role in (1) fine tuning the efficiency of miR160 processing from its precursor transcripts, and subsequently (2) mediating the mode of miR160-directed expression regulation of its *ARF10*, *ARF16*, and *ARF17* target genes [39]. Here, using exogenous application of the synthetic auxin, 2,4-D, we further characterized the regulatory roles played by DRB1 and DRB2 in the miR160 expression module as part of root development. In addition, we performed a bioinformatic assessment of the *cis*-regulatory elements in the putative promoter regions of the loci encoding the molecular components of the miR160 module (Table 1), which showed that the miR160 expression module is an ideal system for molecular analysis of the effect of exogenous auxin application on *Arabidopsis* root system development.

The application of 0.1 μM 2,4-D promoted the development of both the root system and arial tissues of Col-0 plants, indicating that root to shoot auxin signaling was not compromised in Col-0/0.1 μM plants (Figure 1A–E). However, the treatment of Col-0 with 1.0 μM 2,4-D, while enhancing the primary root length and increasing the lateral and adventitious root number, resulted in a significant decrease in the rosette surface area, suggesting that root to shoot auxin signaling was inhibited at the higher 2,4-D concentration. In the roots of Col-0/0.1 μM plants, the expression of *PRE-MIR160A*, *PRE-MIR160B*, and *PRE-MIR160C* was reduced in all cases, as was the expression of *DRB1* (Figure 1F–H,O). In addition, miR160 accumulation was significantly reduced by 10-fold in Col-0/0.1 μM roots (Figure 1I). Thus, the reduced miR160 abundance in Col-0/0.1 μM roots was the result of the combined effect of repressed *MIR160A*, *MIR160B*, and *MIR160C* expression and reduced DRB1-mediated processing of the three miR160 precursors. Of the three miR160 target genes assessed by RT-qPCR (Figure 1L–M), only *ARF16* was significantly upregulated (5.0-fold) in Col-0/0.1 μM roots, which correlated with the decreased abundance of miR160 (Figure 1I,M). ARF16, either on its own or in concert with ARF10, has been shown to promote, or at least maintain, primary root growth in the presence of auxin [12,13,54]. A role for ARF16 in primary root length promotion in Col-0 plants was further confirmed by the phenotypes of Col-0 plants molecularly modified to express a miR160-resistant version of *ARF16* (*mARF16*) [13,39,54]. These previous findings, together with our results shown in Figure 1, suggest that elongation of the primary root in Col-0/0.1 μM plants results from relaxation of miR160-directed regulation of *ARF16* expression.

Compared to Col-0/0.1 μM plants, the higher concentration of 2,4-D had a much greater positive influence on lateral and adventitious root development (Figure 1A,C,D). At the molecular level, application of 1.0 μM 2,4-D only altered *PRE-MIR160B* and *PRE-MIR160C* transcript abundance, with the level of the *PRE-MIR160A* transcript remaining largely unchanged in Col-0/1.0 μM roots, compared to Col-0/0.0 μM roots (Figure 1F–H). These changes in precursor abundance correspond to an overall 2.0-fold decrease in miR160 accumulation in Col-0/1.0 μM roots (Figure 1G–I). Reduced miR160 abundance in Col-0/1.0 μM roots could not be explained by the reduced *DRB2* expression and increased *DRB1* expression (Figure 1O). Reduced *DRB2* expression would minimize its antagonism on DRB1 function to enhance miR160 production [28,35,43], and increased *DRB1* expression would further enhance miR160 processing, both resulting in increased miR160 accumulation. Therefore, the reduced accumulation of miR160 in Col-0/1.0 μM roots is more likely to have resulted from the highly elevated level of abundance of *eTM160-1* (Figure 1J), with this *eTM* sequestering miR160 levels in specific *Arabidopsis* tissues to fine tune the degree of miR160-directed regulation of its *ARF* target genes [47,48].

Of the three miR160 target genes assessed by RT-qPCR in Col-0/1.0 μM roots, altered target gene expression was only observed for *ARF16* and *ARF17* (Figure 1L–N), with *ARF16* expression mildly elevated by 1.4-fold and *ARF17* levels reduced by a similar degree (1.3-fold). In *Arabidopsis*, ARF17 acts as a transcriptional repressor to downregulate the expression of its *ARG*s [12], with *GLYCOSIDE HYDROLASE3*-like (*GH3*-like) genes forming one group of *ARG*s whose expression is negatively regulated by ARF17 [55,56]. GH3-like proteins are responsive to auxin and play a positive regulatory role in primary root elongation and the establishment of lateral root initials, and subsequently, to drive lateral root elongation post their formation [55,56]. Therefore, the mild reduction of *ARF17* gene expression may have released the repressive effect of ARF17 on *GH3*-like gene expression, which in turn directs GH3-like protein-mediated promotion of lateral root formation in Col-0 plants treated with 1.0 μM 2,4-D. It is also important to note here that the correct function of all three miR160-targeted ARFs, including ARF10, ARF16, and ARF17, forms an essential requirement for normal primary and lateral root development in *Arabidopsis* [39,57], with abnormally perceived auxin concentrations leading to defective root system development [39,57,58]. In addition, in Col-0/*mARF16* plants, the primary root length, lateral root number, and lateral root length were all promoted in this *Arabidopsis* transformant line, where the miR160-resistant transgene version of the *ARF16* transcript over-accumulated [39]. Therefore, increased *ARF16* expression, together with decreased *ARF17* expression, likely caused the promotion of lateral root development in Col-0/1.0 μM plants. Of the three *ARF* genes targeted by miR160 in *Arabidopsis*, only *ARF17* has a documented role in adventitious root formation [12,54]. Therefore, although only a mild 1.3-fold reduction was observed for *ARF17* expression in Col-0/1.0 μM roots, this reduction to *ARF17*/ARF17 levels most likely caused the increase in adventitious root number in this sample as ARF17 has been previously demonstrated to repress adventitious root formation in *Arabidopsis* [12,54]. The Col-0/0.1 μM data also support this suggestion, with the adventitious root number only slightly increased in Col-0/0.1 μM plants (*n* = 1.8), compared to untreated control Col-0 plants (*n* = 1.5), in response to the very marginal alteration to *ARF17* expression observed in this sample (Figure 1D,N).

### 4.2. Synthetic Auxin Application Altered drb1 Root Development Due to Molecular Alteration of the miR160 Expression Module

In the *drb1* mutant background, following its treatment with the lower concentration of synthetic auxin, all four of the assessed phenotypic metrics were promoted (Figure 2A–E). In contrast, only the primary root length was altered in *drb1*/1.0 μM plants. When considered together, these findings indicate that root to shoot auxin signaling remained functional in *drb1*/0.1 μM plants, but became compromised in *drb1* plants following their treatment with 1.0 μM 2,4-D. *PRE-MIR160A*, *PRE-MIR160B*, and *PRE-MIR160C* abundance was significantly reduced in *drb1*/0.1 μM roots, and accordingly, miR160 accumulation was significantly elevated by 5.0-fold (Figure 2F–I). In addition, *DRB4* expression was also reduced in *drb1*/0.1 μM roots, while the level of expression of *DRB2* remained largely unchanged (Figure 2P,Q). Together, these transcript abundance trends indicated that in *drb1*/0.1 μM roots, a reduction in DRB4 antagonism of DRB2 function [34] led to more efficient processing of the three miR160 precursor transcripts by DRB2, together with DCL1, in the absence of DRB1 [28,35,36]. In response to increased miR160 abundance, *ARF10* expression was decreased and *ARF16* transcript abundance was increased in *drb1*/0.1 μM roots (Figure 2L,M). Considering that both ARF10 and ARF16 have been assigned roles in regulating primary root development [12,13,54], the *ARF10* and *ARF16* expression trends suggest that elevated *ARF16*/ARF16 abundance could compensate for reduced *ARF10*/ARF10 abundance, which led to the promotion of primary root development in *drb1*/0.1 μM plants. Alteration of the *ARF10* to *ARF16* abundance ratio may have also directed the promotion of lateral root formation in *drb1*/0.1 μM roots (Figure 2C,L,M) with the appropriate function of all three miR160 targeted ARFs demonstrated to be essential for normal root system development in *Arabidopsis* [39,57,58]. Essential regulatory roles for ARF10 and ARF16 in primary and lateral root development in the *drb1* mutant background have been confirmed recently via the *in planta* expression of the *mARF10* and *mARF16* transgenes in *drb1* plants [39]. Moreover, elevation of the abundance of either the *ARF10* or the *ARF16* transcript in either the *drb1*/*mARF10* or *drb1*/*mARF16* transformant line was shown to have a positive influence on both primary root length and lateral root formation [39].

The expression of all three miR160 precursors was also reduced in *drb1*/1.0 μM roots (Figure 2F–H). However, the level of reduction of precursor transcript abundance was less in *drb1*/1.0 μM roots than it was in *drb1*/0.1 μM roots, and accordingly, the level of miR160 over-accumulation was to a lower degree in the roots of *drb1*/1.0 μM plants (Figure 2I). This lower degree of miR160 accumulation enhancement in *drb1*/1.0 μM roots was also likely to be the combined result of (1) reduced *DRB2* expression (Figure 2P), and therefore a reduction in the level of DRB2 protein available to form a functional interaction with DCL1 for miR160 precursor transcript processing [28,35,36], and (2) elevated *eTM160-1* and *eTM160-2* transcript abundance (Figure 2J,K), which would have tightened the posttranscriptional regulation of miR160 accumulation in *drb1*/1.0 μM roots [47,48]. In addition, *ARF10*, *ARF16*, and *ARF17* expression was only mildly altered in *drb1*/1.0 μM roots (Figure 2L–N), indicating that miR160-directed regulation of its target genes became defective in the *drb1* mutant following its treatment with 1.0 μM 2,4-D. Furthermore, this lack of target gene response to altered miR160 abundance was also the likely cause of the lack phenotypic alteration of the *drb1* mutant following its treatment with the higher concentration of synthetic auxin. For example, lateral root formation was highly promoted in *drb1*/0.1 μM plants where the expression of *ARF10* and *ARF16* were both significantly altered (Figure 2C,L,M). However, lateral root development was comparable between the *drb1*/0.0 μM and *drb1*/1.0 μM samples (Figure 2C) where *ARF10*, *ARF16*, and *ARF17* target gene expression was only mildly altered (Figure 2L–N).

### 4.3. Synthetic Auxin Application Altered drb2 Root Development Due to Molecular Alteration of the miR160 Expression Module

The abundance of *PRE-MIR160A*, *PRE-MIR160B*, and *PRE-MIR160C* was reduced in the roots of *drb2*/0.1 μM plants and the abundance of miR160 was significantly elevated (Figure 3F–I). Further, *DRB1* expression was elevated in *drb2*/0.1 μM roots (Figure 3O), indicating that, in the absence of DRB2 antagonism of the DRB1 function, elevated miR160 accumulation was due to enhanced precursor transcript processing efficiency [35,36,37]. In response to elevated miR160 accumulation, *ARF10* and *ARF16* expression was mildly increased by 1.4-fold, and *ARF17* expression was mildly reduced by 1.3-fold (Figure 3L–N). The mildly repressive effect on primary root development in the *drb2*/0.1 μM sample was expected as we have previously shown that molecular modification of the *drb2* mutant via the introduction of the miR160-resistant *mARF10* and *mARF16* transgenes had little influence on primary root length [39]. Interestingly, when these previous findings are considered together with the findings reported here in Figure 3, they suggest that, although *ARF10* and *ARF16* expression was increased in *drb2*/0.1 μM roots, the ability of the ARF10 and ARF16 proteins to direct their known roles in primary root development [12,13,54] is largely lost in the absence of DRB2 function. In contrast to primary root development in *drb2*/0.1 μM plants, lateral root formation was highly promoted (Figure 3A,C). Considering that both ARF10 and ARF16 have previously been assigned positive roles in lateral root development [13,55,56], an elevated level of expression of both *ARF10* and *ARF16* in *drb2*/0.1 μM roots (Figure 3L,M) likely accounted for this phenotypic change (Figure 3A,C).

Lateral root development was promoted to a further extent in the roots of *drb2*/1.0 μM plants (Figure 3A,C). Interestingly, this further promotion of lateral root development occurred in *drb2*/1.0 μM plants even though many of the molecular components of the miR160 expression module were altered to a lesser degree than they were in the roots of *drb2*/0.1 μM plants. More specifically, elevated *DRB1* expression (Figure 3O), and therefore elevated DRB1 protein abundance, only resulted in enhanced processing efficiency of the *PRE-MIR160A* precursor (Figure 3F). In contrast, elevated *PRE-MIR160B* and *PRE-MIR160C* abundance suggested that both precursors were less efficiently processed in the roots of *drb2*/1.0 μM plants (Figure 3G,H). However, although miR160 accumulation was elevated to a lesser extent in *drb2*/1.0 μM roots than in *drb2*/0.1 μM roots (Figure 3I), *ARF10* expression was slightly higher, and *ARF17* expression further reduced in the root system of *drb2*/1.0 μM plants (Figure 3L,N). Interestingly, the higher level of promotion to lateral root development in *drb2*/1.0 μM plants was restricted to the upper region of the root system (Figure 3A). Considering that both ARF10 and ARF16 direct positive regulatory roles in lateral root development in *Arabidopsis* [12,13,54], and that *ARF10* expression was increased to a higher level in the roots of *drb2*/1.0 μM plants than it was in *drb2*/0.1 μM roots, the unique phenotypic response of *drb2* plants to treatment with 1.0 μM 2,4-D may indicate that the positive regulatory role directed by ARF10 in lateral root development in *Arabidopsis* is restricted to the upper region of the root system. As outlined above, of the three *ARF* genes under miR160-directed expression regulation, only ARF17 has been shown to play a role in adventitious root development in *Arabidopsis* [12,54]. Specifically, lateral root formation was repressed in *Arabidopsis* transformants molecularly modified to overexpress *ARF17* via the introduction and *in planta* expression of the miR160-resistant transgene, *mARF17* [12]. As described for the Col-0/1.0 μM sample (Figure 1A,D,N), and stemming from its known role in adventitious root development [12,54], the mild reduction in *ARF17* expression (Figure 3N), and therefore ARF17 protein abundance, most likely caused the significant promotion of adventitious root development in *drb2* plants (Figure 3A,D) following the treatment of this mutant background with the higher concentration of synthetic auxin.

### 4.4. The drb12 Double Mutant Is Largely Defective in Its Ability to Mount a miR160-Directed Response to Treatment with Synthetic Auxin

Of the three root phenotypic measurements assessed in the *drb12* double mutant following its treatment with either 0.1 or 1.0 μM 2,4-D, only adventitious root development was significantly impacted in *drb12*/1.0 μM plants (Figure 4A–D). A lack of phenotypic response by *drb12* plants to treatment with synthetic auxin is somewhat expected as the activity of both DRB1 and DRB2 are required to produce most *Arabidopsis* miRNAs [23,24,25,35,36,37,38], with defective miRNA activity shown to severely impact *Arabidopsis* development [19,20,21,22,31]. Therefore, in addition to defective miR160 production, and miR160-directed regulation of the expression of its target genes, the molecular profile of most *Arabidopsis* miRNA expression modules would be altered in the double mutant [23,24,25,35,36,37,38], which would in turn render *drb12* plants defective in their ability to mount an appropriate miRNA-mediated response to treatment with synthetic auxin. In *drb12*/0.1 μM roots, the *PRE-MIR160A*, *PRE-MIR160B*, and *PRE-MIR160C* transcripts were all reduced in abundance (Figure 4F–H), and the level of miR160 accumulation was also significantly decreased (Figure 4I). In addition, the expression of *DRB4* was also significantly reduced in the roots of *drb12*/0.1 μM plants (Figure 4Q). Considering that our bioinformatic assessment of the *cis*-acting element landscapes of the promotor regions of genes encoding the individual components of the miR160 expression module identified the presence of *ARE*s in the *MIR160A*, *MIR160B*, *MIR160C,* and *DRB4* gene promoter regions, the significantly reduced level of miR160 accumulation in *drb12*/0.1 μM roots was the combined result of (1) repressed *MIR160A*, *MIR160B*, and *MIR160C* gene expression, and (2) the inability of the reduced level of *DRB4*/DRB4 to compensate for the loss of DRB1 and DRB2 function. In response to the significantly reduced abundance of miR160 in *drb12*/0.1 μM roots (Figure 4I), *ARF10* expression was highly increased by 5.0-fold (Figure 5I), and since both ARF10 and ARF16 have been shown to promote primary root development in *Arabidopsis* [12,13,54], elevated *ARF10*/ARF10 abundance was the most likely cause of the slight promotion of primary root length in *drb12*/0.1 μM plants (Figure 4A,B). The significant increase in *ARF10* expression was also the likely cause of increased lateral root numbers in *drb12* plants following their treatment with 0.1 μM 2,4-D, with ARF10 also previously demonstrated to mediate a positive role in lateral root development in *Arabidopsis* [40,43,57,58].

In the roots of *drb12* plants treated with 1.0 μM 2,4-D, and as described for *drb12*/0.1 μM roots, the significant reduction in miR160 accumulation (Figure 4I) was likely the result of repressed *MIR160A* and *MIR160B* gene activity at the transcriptional level (Figure 4F,G), combined with the inability of the significantly reduced level of *DRB4*/DRB4 to compensate for the loss of DRB1 and DRB2 function at the posttranscriptional level (Figure 4Q). In response to reduced miR160 accumulation in *drb12*/1.0 μM roots, *ARF10* expression was significantly elevated and the expression of *ARF16* and *ARF17* mildly increased (Figure 4L–N). Interestingly, although *ARF* target gene expression responded appropriately to reduced miR160 accumulation, the elevated abundance of the *ARF10*, *ARF16*, and *ARF17* target transcripts could not account for the phenotypic response displayed by the root system of the *drb12* double mutant following its treatment with 1.0 μM 2,4-D (Figure 4A–D). For example, ARF10 and ARF16 have both been shown to promote primary root development [12,13,54]. However, although the expression of both miR160 target genes was elevated in *drb12*/1.0 μM roots, the primary root length was reduced. Similarly, ARF17 is the only miR160 target associated with adventitious root development, mediating a repressive role in this context via acting as a transcriptional repressor of the expression of its regulated *ARG*s [12,55,56]. Therefore, considering this documented role for ARF17, the elevated abundance of *ARF17*, albeit mild, would be expected to repress adventitious root formation in *drb12*/1.0 μM plants (Figure 4D,N). However, the average number of adventitious roots per *drb12* plant was significantly increased by the treatment of the *drb12* double mutant with the higher concentration of synthetic auxin (Figure 4A,D). These unexpected findings suggest that, in the absence of DRB1 and DRB2 function, miR160 loses its regulatory ability to appropriately control the expression of its *ARF* target genes for ARF10, ARF16, and ARF17 to continue to mediate their normal functional roles in *Arabidopsis* root system development. Furthermore, considering that most miRNA expression modules would be molecularly altered in the *drb12* double mutant, the phenotypic response displayed by the root system of *drb12* plants following their treatment with 1.0 μM 2,4-D could be the result of molecular alterations to other miRNA expression modules, such as the miR156 and miR167 expression modules, which have been associated with regulating adventitious root development [59,60], and the miR390 and miR847 expression modules involved in regulating lateral root development in *Arabidopsis* [61,62].

### 4.5. In Addition to DRB1 and DRB2, DRB4 Adds an Additional Layer of Regulatory Complexity to the miR160 Expression Module in Arabidopsis Roots

Altered *DRB4* expression in the roots of Col-0 (Figure 1Q), *drb1* (Figure 2Q), *drb2* (Figure 3Q), and *drb12* (Figure 4Q) plants following the treatment of these four *Arabidopsis* lines with synthetic auxin inferred that, in addition to its known requirement for the production of the non-conserved subclass of miRNAs [28,33,34,38], and of tasiARF [52,53], DRB4 is also required to fine tune the rate of production of the conserved miRNA, miR160, in *Arabidopsis* roots. More specifically, in *drb4* and *drb24* roots, *PRE-MIR160A* and *PRE-MIR160B* abundance was reduced and the accumulation of miR160 was significantly elevated (Figure 5F,G,I). This showed that like the documented role played by DRB2 in regulating the rate of production of specific miRNA subsets in *Arabidopsis* [35,36,37,38], DRB4 also plays a secondary role in regulating the rate of miR160 production, and that this occurs via DRB4 antagonism of DRB1 function in the DCL1/DRB1 partnership to influence the efficiency of miR160 precursor transcript processing. In addition, DRB4 has previously been shown to be antagonistic towards the role of DRB2 in the production of the non-conserved subclass of miRNAs and other subspecies of siRNAs in *Arabidopsis* [34,38]. Here, the significantly elevated abundance of *PRE-MIR160A*, *PRE-MIR160B*, and *PRE-MIR160C* in the roots of *drb14* plants (Figure 5F–H), together with elevated miR160 accumulation in the roots of this double mutant (Figure 5I), revealed that DRB4 is also antagonistic towards the secondary role played by DRB2 to fine tune the rate of miR160 production in *Arabidopsis* roots. Furthermore, DRB4 antagonism of DRB1 and DRB2 function in miR160 production appears to occur at the transcriptional level, as evidenced by the elevated level of *DRB1* expression in *drb24* roots, and increased *DRB2* expression in *drb4* and *drb14* roots (Figure 5O,P). In *drb14* roots, the expression levels of *ARF10*, *ARF16*, and *ARF17* were all significantly elevated (Figure 5L–N). Considering the known roles of ARF10, ARF16, and ARF17 in regulating primary, lateral, and adventitious root development in *Arabidopsis* [12,13,40,44,54,55,56,57,58], these miR160 target gene expression trends would be expected to promote primary and lateral root development and to repress adventitious root development in the *drb14* double mutant, respectively. However, opposing and therefore unexpected root phenotypes were observed for *drb14* plants. Moreover, the primary root length was reduced, the lateral root number was mildly repressed, and adventitious root formation was highly promoted in the *drb14* double mutant (Figure 4A–D). When considered together, these results suggest that the functional roles played by the *ARG*s in root development which are under the transcriptional control of ARF10, ARF16, and ARF17 become defective in the absence of DRB1 and DRB4 function in miR160 production. This suggestion is further supported by comparison of the *ARF10*, *ARF16*, and *ARF17* expression trends in *drb24* roots (Figure 5L–N) and the root phenotypes displayed by this double mutant background (Figure 5A–D). More specifically, this comparison again suggests that the ability of the *ARG*s under the transcriptional control of ARF10, ARF16, and ARF17 to direct their functional roles in controlling *Arabidopsis* root development is lost in the absence of the involvement of DRB2 and DRB4 in miR160 production.

## 5. Conclusions

In this study, we molecularly profiled the miR160 expression module in the roots of Col-0, *drb1*, *drb2*, and *drb12* plants following the treatment of these four *Arabidopsis* lines with 2,4-D. In the roots of 0.1 μM 2,4-D treated Col-0, *drb1*, and *drb2* plants, the root phenotype alterations displayed by these three *Arabidopsis* lines were associated with the altered accumulation level of miR160, and the changes in the level of expression of its target genes, *ARF10*, *ARF16*, and *ARF17*. More specifically, altered *ARF10* and/or *ARF16* expression was associated with promoted primary and lateral root development in Col-0, *drb1*, and *drb2* plants following their treatment with 0.1 μM 2,4-D, and, similarly, altered *ARF17* expression was associated with promoted adventitious root development in Col-0/0.1 μM, *drb1*/0.1 μM, and *drb2*/0.1 μM plants. In contrast, such an association could not be established for the *drb12* double mutant following its treatment with either 0.1 or 1.0 μM 2,4-D, nor could such a relationship be established in Col-0, *drb1*, and *drb2* plants following their treatment with the higher concentration of 2,4-D. In addition, by performing the same molecular profiling exercise of the miR160 expression module in the root systems of *drb4*, *drb14*, and *drb24* plants, we identified a previously unknown role for DRB4 in regulating miR160 production in *Arabidopsis*. Taken together, the molecular data presented here show that the correct function of DRB1, DRB2, and DRB4 is required to control miR160 production, and subsequently, to appropriately regulate *ARF10*, *ARF16*, and *ARF17* target gene expression, for normal root system development in *Arabidopsis*.

## Data Availability

The original contributions presented in the study are included in the article/Supplementary Material, further inquiries can be directed to the corresponding author.

## Supplementary Materials

The following supporting information can be downloaded at: https://www.mdpi.com/article/10.3390/genes15121648/s1, Table S1: Sequences of the DNA oligonucleotides used in this study.

## References

[1] Ruegger M., Dewey E., Gray W.M., Hobbie L., Turner J., Estelle M. (1998). The TIR1 protein of *Arabidopsis* functions in auxin response and is related to human SKP2 and yeast grr1p. Genes.

[2] Guilfoyle T.J., Hagen G. (2007). Auxin response factors. Curr. Opin. Plant Biol..

[3] Kepinski S., Leyser O. (2004). Auxin-induced SCFTIR1-Aux/IAA interaction involves stable modification of the SCFTIR1 complex. Proc. Natl. Acad. Sci. USA.

[4] Reed J.W. (2001). Roles and activities of Aux/IAA proteins in *Arabidopsis*. Trends Plant Sci..

[5] Luo J., Zhou J.J., Zhang J.Z. (2018). Aux/IAA Gene Family in Plants: Molecular Structure, Regulation, and Function. Int. J. Mol. Sci..

[6] Guilfoyle T.J., Hagen G. (2001). Auxin response factors. J. Plant Growth Reg..

[7] Tiwari S.B., Hagen G., Guilfoyle T.J. (2003). The roles of auxin response factor domains in auxin-responsive transcription. Plant Cell.

[8] Boer D.R., Freire-Rios A., van den Berg W.A., Saaki T., Manfield I.W., Kepinski S., Lopez-Vidrieo I., Franco-Zorrilla J.M., de Vries S.C., Solano R. (2014). Structural basis for DNA binding specificity by the auxin-dependent ARF transcription factors. Cell.

[9] Hardtke C.S., Berleth T. (1998). The *Arabidopsis* gene *MONOPTEROS* encodes a transcription factor mediating embryo axis formation and vascular development. EMBO J..

[10] Hamann T., Benkova E., Baurle I., Kientz M., Jurgens G. (2002). The *Arabidopsis BODENLOS* gene encodes an auxin response protein inhibiting MONOPTEROS-mediated embryo patterning. Genes Dev..

[11] Ellis C.M., Nagpal P., Young J.C., Hagen G., Guilfoyle T.J., Reed J.W. (2005). AUXIN RESPONSE FACTOR1 and AUXIN RESPONSE FACTOR2 regulate senescence and floral organ abscission in *Arabidopsis thaliana*. Development.

[12] Mallory A.C., Bartel D.P., Bartel B. (2005). MicroRNA-directed regulation of Arabidopsis AUXIN RESPONSE FACTOR17 is essential for proper development and modulates expression of early auxin response genes. Plant Cell.

[13] Wang J.W., Wang L.J., Mao Y.B., Cai W.J., Xue H.W., Chen X.Y. (2005). Control of root cap formation by microRNA-targeted auxin response factors in *Arabidopsis*. Plant Cell.

[14] Ru P., Xu L., Ma H., Huang H. (2006). Plant fertility defects induced by the enhanced expression of microRNA167. Cell Res..

[15] Wu M.F., Tian Q., Reed J.W. (2006). *Arabidopsis* microRNA167 controls patterns of *ARF6* and *ARF8* expression, and regulates both female and male reproduction. Development.

[16] Williams L., Carles C.C., Osmont K.S., Fletcher J.C. (2005). A database analysis method identifies an endogenous *trans*-acting short-interfering RNA that targets the *Arabidopsis ARF2*, *ARF3*, and *ARF4* genes. Proc. Natl. Acad. Sci. USA.

[17] Hunter C., Willmann M.R., Wu G., Yoshikawa M., de la Luz Gutierrez-Nava M., Poethig S.R. (2005). *Trans*-acting siRNA-mediated repression of ETTIN and ARF4 regulates heteroblasty in *Arabidopsis*. Development.

[18] Finet C., Fourquin C., Vinauger M., Berne-Dedieu A., Chambrier P., Paindavoine S., Scutt C.P. (2010). Parallel structural evolution of auxin response factors in the angiosperms. Plant J..

[19] Vidal E.A., Araus V., Lu C., Parry G., Green P.J., Coruzzi G.M., Gutierrez R.A. (2010). Nitrate-responsive miR393/AFB3 regulatory module controls root system architecture in *Arabidopsis thaliana*. Proc. Natl. Acad. Sci. USA.

[20] Liu D., Song Y., Chen Z., Yu D. (2009). Ectopic expression of miR396 suppresses GRF target gene expression and alters leaf growth in *Arabidopsis*. Physiol. Plant.

[21] Song J.B., Huang S.Q., Dalmay T., Yang Z.M. (2012). Regulation of leaf morphology by microRNA394 and its target LEAF CURLING RESPONSIVENESS. Plant Cell Physiol..

[22] Vazquez F., Gasciolli V., Crété P., Vaucheret H. (2004). The nuclear dsRNA binding protein HYL1 is required for microRNA accumulation and plant development, but not posttranscriptional transgene silencing. Curr. Biol..

[23] Kurihara Y., Takashi Y., Watanabe Y. (2006). The interaction between DCL1 and HYL1 is important for efficient and precise processing of pri-miRNA in plant microRNA biogenesis. RNA.

[24] Song L., Han M.H., Lesicka J., Fedoroff N. (2007). *Arabidopsis* primary microRNA processing proteins HYL1 and DCL1 define a nuclear body distinct from the Cajal body. Proc. Natl. Acad. Sci. USA.

[25] Han M.H., Goud S., Song L., Fedoroff N. (2004). The *Arabidopsis* double-stranded RNA-binding protein HYL1 plays a role in microRNA-mediated gene regulation. Proc. Natl. Acad. Sci. USA.

[26] Fang Y., Spector D.L. (2007). Identification of nuclear dicing bodies containing proteins for microRNA biogenesis in living *Arabidopsis* plants. Curr. Biol..

[27] Fujioka Y., Utsumi M., Ohba Y., Watanabe Y. (2007). Location of a possible miRNA processing site in SmD3/SmB nuclear bodies in *Arabidopsis*. Plant Cell Physiol..

[28] Eamens A.L., Smith N.A., Curtin S.J., Wang M.B., Waterhouse P.M. (2009). The *Arabidopsis thaliana* double-stranded RNA binding protein DRB1 directs guide strand selection from microRNA duplexes. RNA.

[29] Fagard M., Boutet S., Morel J.B., Bellini C., Vaucheret H. (2000). AGO1, QDE-2, and RDE-1 are related proteins required for posttranscriptional gene silencing in plants, quelling in fungi, and RNA interference in animals. Proc. Natl. Acad. Sci. USA.

[30] Morel J.B., Godon C., Mourrain P., Béclin C., Boutet S., Feuerbach F., Proux F., Vaucheret H. (2002). Fertile hypomorphic *ARGONAUTE1* (*ago1*) mutants impaired in post-transcriptional gene silencing and virus resistance. Plant Cell.

[31] Vaucheret H., Vazquez F., Crété P., Bartel D.P. (2004). The action of ARGONAUTE1 in the miRNA pathway and its regulation by the miRNA pathway are crucial for plant development. Genes Dev..

[32] Baumberger N., Baulcombe D.C. (2005). *Arabidopsis* ARGONAUTE1 is an RNA Slicer that selectively recruits microRNAs and short interfering RNAs. Proc. Natl. Acad. Sci. USA.

[33] Rajagopalan R., Vaucheret H., Trejo J., Bartel D.P. (2006). A diverse and evolutionarily fluid set of microRNAs in *Arabidopsis thaliana*. Genes Dev..

[34] Pélissier T., Clavel M., Chaparro C., Pouch-Pélissier M.N., Vaucheret H., Deragon J.M. (2011). Double-stranded RNA binding proteins DRB2 and DRB4 have an antagonistic impact on polymerase IV-dependent siRNA levels in *Arabidopsis*. RNA.

[35] Eamens A.L., Kim K.W., Curtin S.J., Waterhouse P.M. (2012). DRB2 is required for microRNA biogenesis in *Arabidopsis thaliana*. PLoS ONE.

[36] Eamens A.L., Kim K.W., Waterhouse P.M. (2012). DRB2, DRB3 and DRB5 function in a non-canonical microRNA pathway in *Arabidopsis thaliana*. Plant Signal. Behav..

[37] Reis R.S., Hart-Smith G., Eamens A.L., Wilkins M.R., Waterhouse P.M. (2015). Gene regulation by translational inhibition is determined by Dicer partnering proteins. Nat. Plants.

[38] Curtin S.J., Watson J.M., Smith N.A., Eamens A.L., Blanchard C.L., Waterhouse P.M. (2008). The roles of plant dsRNA-binding proteins in RNAi-like pathways. FEBS Lett..

[39] Zimmerman K., Pegler J.L., Oultram J.M.J., Collings D.A., Wang M.B., Grof C.P.L., Eamens A.L. (2024). Molecular manipulation of the miR160/*AUXIN RESPONSE FACTOR* expression module impacts root development in *Arabidopsis thaliana*. Genes.

[40] Overvoorde P., Fukaki H., Beeckman T. (2010). Auxin control of root development. Cold Spring Harb. Perspect. Biol..

[41] Oono Y., Ooura C., Rahman A., Aspuria E.T., Hayashi K., Tanaka A., Uchimiya H. (2003). p-Chlorophenoxyisobutyric acid impairs auxin response in *Arabidopsis* root. Plant Physiol..

[42] Poupart J., Waddell C.S. (2000). The rib1 mutant is resistant to indole-3-butyric acid, an endogenous auxin in *Arabidopsis*. Plant Physiol..

[43] Rahman A., Nakasone A., Chhun T., Ooura C., Biswas K.K., Uchimiya H., Tsurumi S., Baskin T.I., Tanaka A., Oono Y. (2006). A small acidic protein 1 (SMAP1) mediates responses of the *Arabidopsis* root to the synthetic auxin 2,4-dichlorophenoxyacetic acid. Plant J..

[44] de Reuille P.B., Bohn-Courseau I., Ljung K., Morin H., Carraro N., Godin C., Traas J. (2006). Computer simulations reveal properties of the cell-cell signaling network at the shoot apex in *Arabidopsis*. Proc. Natl. Acad. Sci. USA.

[45] García M.J., Romera F.J., Stacey M.G., Stacey G., Villar E., Alcántara E., Pérez-Vicente R. (2013). Shoot to root communication is necessary to control the expression of iron-acquisition genes in Strategy I plants. Planta.

[46] Taylor-Teeples M., Lanctot A., Nemhauser J.L. (2016). As above, so below: Auxin’s role in lateral organ development. Dev. Biol..

[47] Wu H.J., Wang Z.M., Wang M., Wang X.J. (2013). Widespread long noncoding RNAs as endogenous target mimics for microRNAs in plants. Plant Physiol..

[48] Wang M., Wu H.J., Fang J., Chu C., Wang X.J. (2017). A long noncoding RNA involved in rice reproductive development by negatively regulating osa-miR160. Sci. Bull..

[49] Lescot M., Déhais P., Thijs G., Marchal K., Moreau Y., Van de Peer Y., Rouzé P., Rombauts S. (2002). PlantCARE, a database of plant *cis*-acting regulatory elements and a portal to tools for in silico analysis of promoter sequences. Nucleic Acids Res..

[50] Higo K., Ugawa Y., Iwamoto M., Korenaga T. (1999). Plant cis-acting regulatory DNA elements (PLACE) database: 1999. Nucleic Acids Res..

[51] Davuluri R.V., Sun H., Palaniswamy S.K., Matthews N., Molina C., Kurtz M., Grotewold E. (2003). AGRIS: *Arabidopsis* gene regulatory information server, an information resource of *Arabidopsis cis*-regulatory elements and transcription factors. BMC Bioinform..

[52] Nakazawa Y., Hiraguri A., Moriyama H., Fukuhara T. (2007). The dsRNA-binding protein DRB4 interacts with the Dicer-like protein DCL4 in vivo and functions in the trans-acting siRNA pathway. Plant Mol. Biol..

[53] Adenot X., Elmayan T., Lauressergues D., Boutet S., Bouché N., Gasciolli V., Vaucheret H. (2006). DRB4-dependent *TAS3 trans*-acting siRNAs control leaf morphology through AGO7. Curr. Biol..

[54] Couzigou J.M., Combier J.P. (2016). Plant microRNAs: Key regulators of root architecture and biotic interactions. New Phytol..

[55] Nakazawa M., Yabe N., Ichikawa T., Yamamoto Y.Y., Yoshizumi T., Hasunuma K., Matsui M. (2001). DFL1, an auxin-responsive GH3 gene homologue, negatively regulates shoot cell elongation and lateral root formation, and positively regulates the light response of hypocotyl length. Plant J..

[56] Takase T., Nakazawa M., Ishikawa A., Kawashima M., Ichikawa T., Takahashi N., Shimada H., Manabe K., Matsui M. (2004). ydk1-D, an auxin-responsive GH3 mutant that is involved in hypocotyl and root elongation. Plant J..

[57] Zhao Y. (2010). Auxin biosynthesis and its role in plant development. Ann. Rev. Plant Biol..

[58] Leyser O. (2002). Molecular genetics of auxin signaling. Ann. Rev. Plant Biol..

[59] Gutierrez L., Bussell J.D., Pacurar D.I., Schwambach J., Pacurar M., Bellini C. (2009). Phenotypic plasticity of adventitious rooting in *Arabidopsis* is controlled by complex regulation of *AUXIN RESPONSE FACTOR* transcripts and microRNA abundance. Plant Cell.

[60] Xu M., Hu T., Zhao J., Park M.Y., Earley K.W., Wu G., Yang L., Poethig R.S. (2016). Developmental Functions of miR156-Regulated *SQUAMOSA PROMOTER BINDING PROTEIN-LIKE* (*SPL*) Genes in *Arabidopsis thaliana*. PLoS Genet..

[61] Yoon E.K., Yang J.H., Lim J., Kim S.H., Kim S.K., Lee W.S. (2010). Auxin regulation of the microRNA390-dependent transacting small interfering RNA pathway in *Arabidopsis* lateral root development. Nucleic Acids Res..

[62] Wang J.J., Guo H.S. (2015). Cleavage of *INDOLE-3-ACETIC ACID INDUCIBLE28* mRNA by microRNA847 upregulates auxin signaling to modulate cell proliferation and lateral organ growth in *Arabidopsis*. Plant Cell.

