# Supplementary material for: The Arabidopsis thaliana Double-Stranded RNA Binding Proteins DRB1 and DRB2 Are Required for miR160-Mediated Responses to Exogenous Auxin
Source: Genes (Basel). 2024 Dec 21;15(12):1648. doi: 10.3390/genes15121648 (PMC11675975; doi:10.3390/genes15121648)
Supplement: Supplementary file 1 [file genes-15-01648-s001.zip › genes-3368541-supplementary.pdf]

Table S1. DNA oligonucleotides used in this study.

Name	Target	Sequence (5′ to 3′)
Stem-Loop RT-qPCR Primers		
pSNO101-F	sno101	CTTCACAGGTAAGTTCGCTTG
pSNO101-R		AGCATCAGCAGACCAGTAGTT
pmiR160-cDNA-R	miR160	GTCGTATCCAGTGCAGGGTCCGAGGTATTCGCACTGGATACGACTGGCAT
pmiR160-cDNA-F		GCTGCCTGGCTCCCTGT
pGeneric-STL		CCAGTGCAGGGTCCGAGGTA
RT-qPCR Primers		
pMIR160A-F	*PRE-MIR160A*	ATATGCTGAGCCCATCGAGTATCG
pMIR160A-R		ATGCATGGCTCCTCATACGCC
pMIR160B-F	*PRE-MIR160B*	GCCACAAGAAAACATCGATTTAGTTTC
pMIR160B-R		TGCTTGACTACTCTGTACGCCA
pMIR160C-F	*PRE-MIR160C*	CCACGAGTGGATACCGATTTTG
pMIR160C-R		GCTTGACTCCTTGTACGCCAC
pETM160-1F	*eTM160-1*	TCTTCAGAGATGGCCTGACGA
pETM160-1R		AATCGTAATCCTAATCAGTGTT
pETM160-2F	*eTM160-2*	ACCGGACTGTCAGTGCTTGAT
pETM160-2R		TTCGCAAATGTCACTCCAAAA
pARF10-F	*ARF10*	CGGTTTTTGGAAGAAGAGGCGG
pARF10-R		GCGTCCAACATCCTCAGATTCCAT
pARF16-F	*ARF16*	AACTTTCTCCTTCTCTCGGTCTCCG
pARF16-R		AGCTTGCCGAACAATACAATATGGG
pARF17-F	*ARF17*	CGAGTCAAGATGGCTATGGA
pARF17-R		CATCCCATGTGATCTGAAGC
pDRB1-F	*DRB1*	ATGACCTCCACTGATGTTTCC
pDRB1-R		TGCTAATTCCCGGAGAGC
pDRB2-F	*DRB2*	ATGTATAAGAACCAGCTACAAGAGTTG
pDRB2-R		CAGCAGCAGAGTGTTCAGC
pDRB4-F	*DRB4*	AAATGGGAACTCGAACCAGA
pDRB4-R		CCACCTTGGAAGAAGGTTGA
pEF1-A-F	*EF1-α*	TGAGCACGCTCTTCTTGCTTTCA
pEF1-A-R		GGTGGTGGCATCCATCTTGTTACA
